# Artificial intelligence-assisted multiscale lung modeling to predict alveolar septal wall stress

**DOI:** 10.1016/j.actbio.2025.11.030

**Published:** 2025-11-27

**Authors:** Sunder Neelakantan, Mostafa K. Ismail, Nikhil Kadivar, Elizabeth McGinn, Luis Loza, Kyle J. Myers, Bradford J. Smith, Rahim Rizi, George Karniadakis, Reza Avazmohammadi

**Affiliations:** a Department of Biomedical Engineering, Texas A&M University, College Station, TX, USA; b Department of Bioengineering, University of Pennsylvania, Philadelphia, PA, USA; c School of Engineering, Brown University, Providence, RI, USA; d Department of Pediatric Pulmonary and Sleep Medicine, School of Medicine, University of Colorado, Aurora, CO, USA; e Hagler Institute for Advanced Study, Texas A&M University, College Station, TX, USA; f Department of Bioengineering, University of Colorado Denver | Anschutz Medical Campus, Aurora, CO, USA; g Department of Radiology, Perelman School of Medicine, University of Pennsylvania, Philadelphia, PA, USA; h J. Mike Walker ‘66 Department of Mechanical Engineering, Texas A&M University, College Station, TX, USA; i Department of Cardiovascular Sciences, Houston Methodist Academic Institute, Houston, TX, USA

**Keywords:** Lung parenchyma, Alveolar septal wall stress, Artificial intelligence, Radiation-induced lung injury

## Abstract

Injuries to the lung parenchyma, such as radiation-induced lung injury (RILI), can lead to heterogeneous ventilation and reduced lung function. The biomechanical drivers behind the onset and progression of such lung injuries are poorly understood. In this study, we developed a method based on tissue mechanical modeling and machine learning to estimate in-vivo alveolar septal wall stress, to understand the effect of parenchymal biomechanics on the onset and progression of lung injuries. Representative tissue elements (RTEs) of the lung parenchyma were reconstructed from X-ray microtomography imaging. A generative adversarial network (GAN) model was used to create synthetic RTEs to enhance the training data for an artificial neural network (ANN). Feature analysis indicated that the GAN model generated synthetic RTEs with features similar to those of the segmented RTEs. Finite element simulations were performed on both the segmented and synthetic RTEs and used to train the ANN. Stretch and geometric features served as inputs, and RTE stresses were the outputs. The ANN’s testing accuracy was 61.6% before and 84.0% after including the synthetic RTEs. We used the ANN to investigate the evolution of stress in a rodent model of RILI. Reduced stress was observed in the lung tissue at 3-month post-radiation, indicating pneumonitis, followed by elevated stress at 5-month post-radiation, suggesting regional fibrosis. Further, stress heterogeneity at the 5-month timepoint indicated the presence of volutrauma. Overall, these results suggest that regional biomechanical markers can be used for early diagnosis and assessment of subclinical lung injuries that existing global measures cannot detect.

## 1. Introduction

Lung injuries such as acute respiratory distress syndrome (ARDS) [1, 2], acute lung injury (ALI), and radiation-induced lung injury (RILI) [3, 4] can lead to heterogeneous alterations in lung biomechanics. These alterations can impair gas exchange and, in severe cases, require mechanical ventilation to maintain life. However, mechanical ventilation can injure the remaining healthy lung regions and worsen the injured regions, causing ventilator-induced lung injury (VILI) [5–7]. The onset and progression of lung injuries depend both on biomechanical drivers and pathophysiological processes. Although the pathophysiological processes associated with lung injury have been previously studied [1,4], its biomechanical drivers of the onset and progression are poorly understood.

For clinical applications, organ-level measurements, such as X-ray imaging [8] and spirometry [9], are the most widely used methods for diagnosing lung injury and estimating lung function. However, spirometry measurements are averaged across both lungs (lacking spatial resolution), while imaging methods, such as X-rays, provide a snapshot of a single instance of respiration (lacking temporal resolution). Such methods do not provide sufficient information to understand and predict the progression of lung injury, especially given their heterogeneous nature. For further spatial and temporal information, methods such as computed tomography (CT) [10,11] and magnetic resonance imaging (MRI) [12,13] have been used. CT imaging provides a 3-dimensional view of the lungs. It can be useful in analyzing the size and position of atelectasis and edema regions, which can be used to infer inflammation regions. Similarly, MRI can produce images with resolution similar to CT, but without ionizing radiation. MRI with hyperpolarized gases as contrast agents has been used to evaluate regional ventilation by estimating gas distribution in the lungs [12,13]. In addition to static CT and MR imaging, studies have used dynamic CT (4DCT) imaging in combination with image registration to analyze regional mesoscale displacement and deformation in the lungs during respiration [14–17]. This mesoscale deformation can be used to estimate and predict regions at risk for volutrauma (overdistension) and atelectrauma (damage from repeated collapse and reopening of the alveoli and small airways). Although estimating the regional kinematic behavior of parenchymal tissue during respiration has been explored, there is currently no method to estimate regional stresses in alveolar septal walls.

Our objective in this study was to introduce alveolar septal wall stress (SWS) as a biomarker for monitoring lung parenchymal health. We aimed to develop a method to estimate SWS in vivo from mesoscale strains, which enabled us to investigate the effect of parenchymal biomechanics on the onset and spread of lung injuries. Estimating regional SWS from regional deformation is challenging due to the complex microstructure of parenchymal tissue. In-vivo [14–17] and ex-vivo [18,19] studies have reported variations in regional strains in the lungs during respiration. Studies have reported that mesoscale strains, as estimated by image registration, are smaller than those experienced by alveolar septal walls [20]. This indicates the need for multiscale modeling [21], incorporating parenchymal microstructure [20,22,23], constitutive parenchymal tissue models [24,25], and mesoscale strains [16,26] to accurately estimate SWS. However, finite element (FE) models of the entire lung that incorporate the complex parenchymal microstructure would be unfeasible due to the lack of clinical methods to capture the parenchymal microstructure in vivo and the computational expenses of simulating a microstructural model of the entire lung. Machine learning (ML) provides an alluring avenue to overcome these challenges. ML has emerged as a powerful tool to supplement mechanistic modeling by training the ML model on synthetic data generated from FE simulations [27,28]. Although training an ML model can require significant time and computational resources, implementing ML models is relatively easy and can be applied directly to clinical measurements. This improved feasibility and ease of use can significantly enhance the potential for clinical translation.

**Fig. 1.**
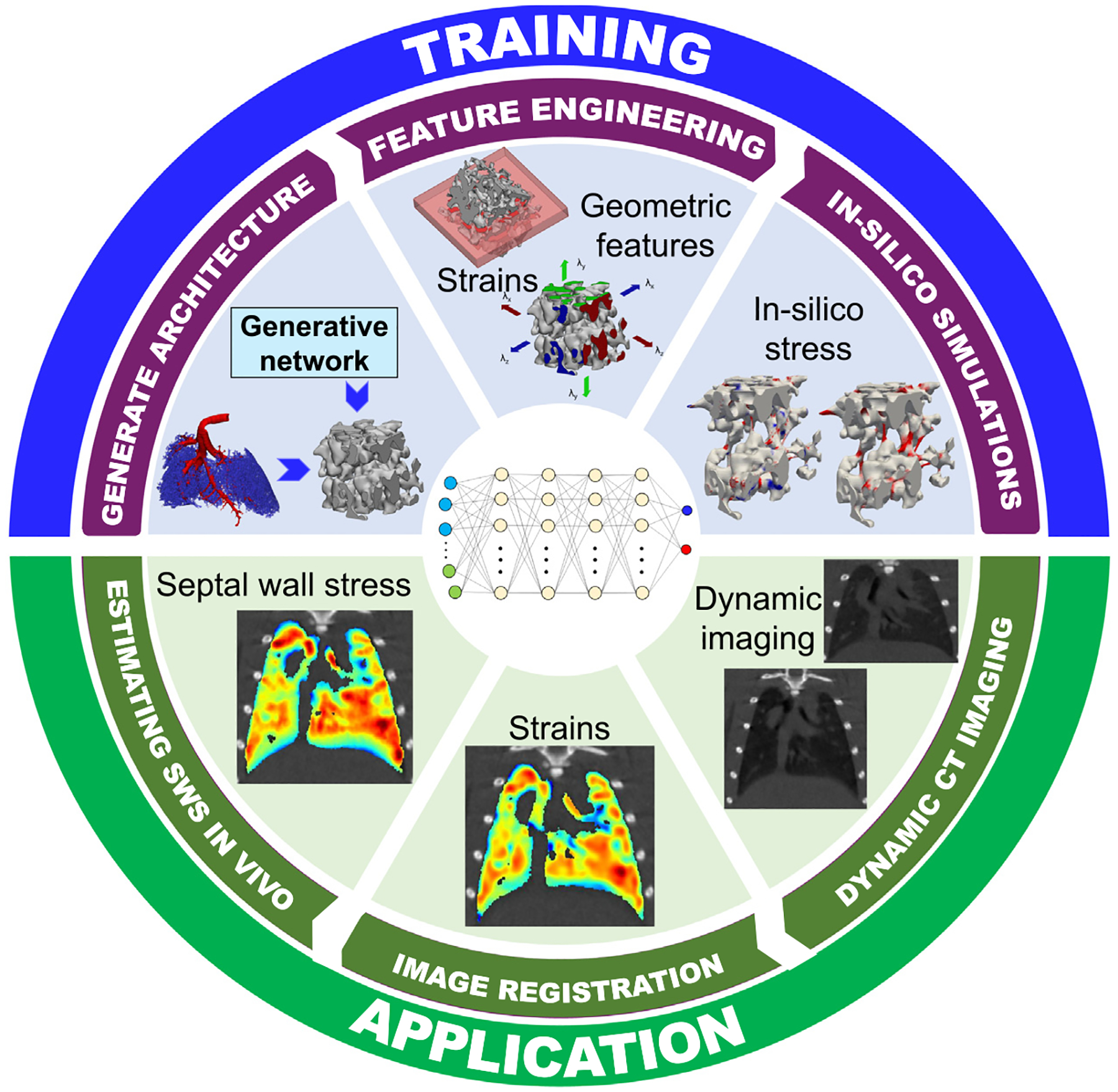
Overview of the proposed workflow presented in this study. The machine learning model consists of a neural network trained on in-silico finite element (FE) simulations of alveolar microstructures segmented from X-ray microtomography images. The model is then applied to dynamic lung images, combined with strains obtained via image registration, to estimate alveolar septal wall stress in vivo.

In this study, X-ray micro-computed tomography (μCT) scans of a rat lung were segmented to reconstruct the parenchymal microstructure. We isolated small cubic regions as the smallest representative elements, referred to as representative tissue elements (RTEs). The combination of RTEs reconstructed from different regions of both lungs can comprehensively capture the behavior of the entire lungs (Fig. 1). In addition to segmenting RTEs, we used a generative adversarial network (GAN) to generate synthetic RTEs, enhancing the variation in RTE geometries used by the ML model (Fig. 1). We performed in-silico FE simulations of both the segmented and synthetic RTEs and used the results to train an ML model. The boundary conditions and geometric features were used as inputs, and RTE stresses were the model’s output. The ML model was used to infer local tissue stress based on voxel intensity (Hounsfield units) and the behavior of similar RTEs.

We applied the ML model to understand the evolution of SWS and strains in a rat model of RILI. To understand the relationship between SWS and lung injuries, we aimed to study the evolution of parenchymal kinematics and SWS in a rodent model of RILI. RILI is a complication associated with thoracic radiotherapy (RT) [29]. RT is used as a treatment or palliative option for malignant and non-malignant disorders [30]. RILI can lead to radiation pneumonitis and, subsequently, radiation fibrosis. RILI was chosen for its ability to modulate severity with radiation dose in follow-up studies. In the current study, dynamic CT scans acquired at the pre-radiation timepoint and at the 1, 3, and 5-month post-radiation timepoints were used to estimate the evolution of regional kinematic behavior using image registration. Next, we used the trained ML model to estimate the evolution of the regional SWS (Fig. 1). We expect the ML model presented in this study to serve as a framework to investigate the evolution of parenchymal kinetics in lung injuries in clinical settings and serve as an advanced biomarker for the onset and progression of lung injuries.

## 2. Methods

All procedures were performed in accordance with the Guide for the Care and Use of Laboratory Animals published by the US National Institutes of Health. Approval for procedures on animal subjects was obtained from the Institutional Animal Care and Use Committees (IACUCs) of the University of Colorado and the University of Pennsylvania.

### 2.1. Reconstruction of representative tissue element from μCT imaging

**Fig. 2.**
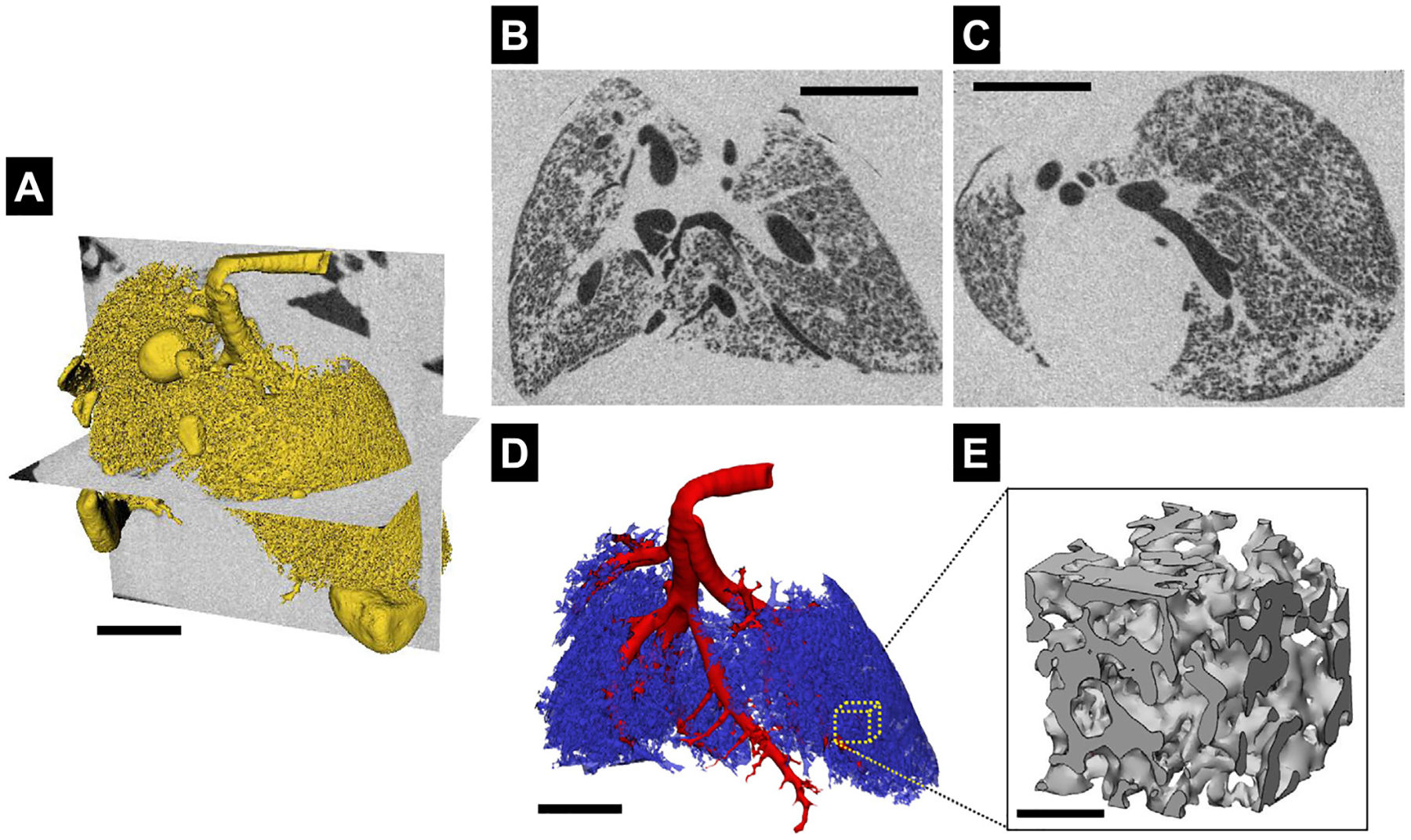
(**A**) X-ray microtomography imaging and segmentation of fixed rat lungs in the (**B**) frontal view and (**C**) transverse view. (**D**) Reconstructed geometry of the lungs, parenchyma (blue), and airways (red). (**E**) An isolated cube selected to be the representative tissue element (RTE). The gray region represents the alveolar septal walls. Scale bars in (**A**)-(**D**) represent 5 mm. Scale bar in (**E**) represents 1 mm.

To obtain the parenchymal microstructure of the lungs, we used the lungs from healthy Sprague–Dawley rats (n=3). After sacrifice, the trachea was filled with 10% formalin and held at a constant pressure head of 10 cm H_2_O to fix the lungs in an inflated state. The lungs were isolated and μCT was used to image the lungs (Figs. 2A–C) with a voxel size of 150μm. The resulting μCT images were used to segment the air space in the lungs (Fig. 2D). Cubic regions with 2 mm sides were isolated from this airspace, and the empty space in these cubes was reconstructed to serve as the alveolar septal walls (Fig. 2D). The RTEs were subsequently meshed using Materialize 3-Matic. Any RTEs with a tissue percentage (by volume) greater than 50% were discarded. In addition, any RTEs with obvious airways were discarded. Finally, RTEs that contained floating sections that were unconnected to the remaining RTEs were discarded during the FE simulations. After discarding all the unsuitable RTEs, 300 total RTEs were obtained from the μCT images of the lungs.

### 2.2. In-silico FE simulations of the RTE elements

In this study, we have chosen to represent all first-order tensors (vectors) using a bold variable with a single overline, such as $\bar{A}$. Similarly, we have chosen to represent all second-order tensors (matrices) using a bold variable with a double overline, such as $\bar{\bar{B}}$. Variables without overlines are considered scalars. To estimate parenchymal kinematics, displacement $\left(\bar{u}=\left[u_{x},\;u_{y},\;u_{z}\right]\right)$ was used to estimate the deformation gradient $\left(\bar{\bar{F}}\right)$ as

(1) $$\bar{\bar{F}}=\bar{\bar{I}}+\left[\begin{array}{lll} \frac{\partial u_{x}}{\partial X} & \frac{\partial u_{x}}{\partial Y} & \frac{\partial u_{x}}{\partial Z}\\ \frac{\partial u_{y}}{\partial X} & \frac{\partial u_{y}}{\partial Y} & \frac{\partial u_{y}}{\partial Z}\\ \frac{\partial u_{z}}{\partial X} & \frac{\partial u_{z}}{\partial Y} & \frac{\partial u_{z}}{\partial Z} \end{array}\right],$$

where $\bar{\bar{I}}$ is the identity matrix. The deformation gradient $\left(\bar{\bar{F}}\right)$ was used to estimate the Cauchy strain $\left(\bar{\bar{C}}\right)$ and the Green–Lagrange strain $\left(\bar{\bar{E}}\right)$.

(2) $$\bar{\bar{C}}={\bar{\bar{F}}}^{T}\bar{\bar{F}},$$

(3) $$\bar{\bar{E}}=\frac{\bar{\bar{C}}-\bar{\bar{I}}}{2}.$$

**Fig. 3.**
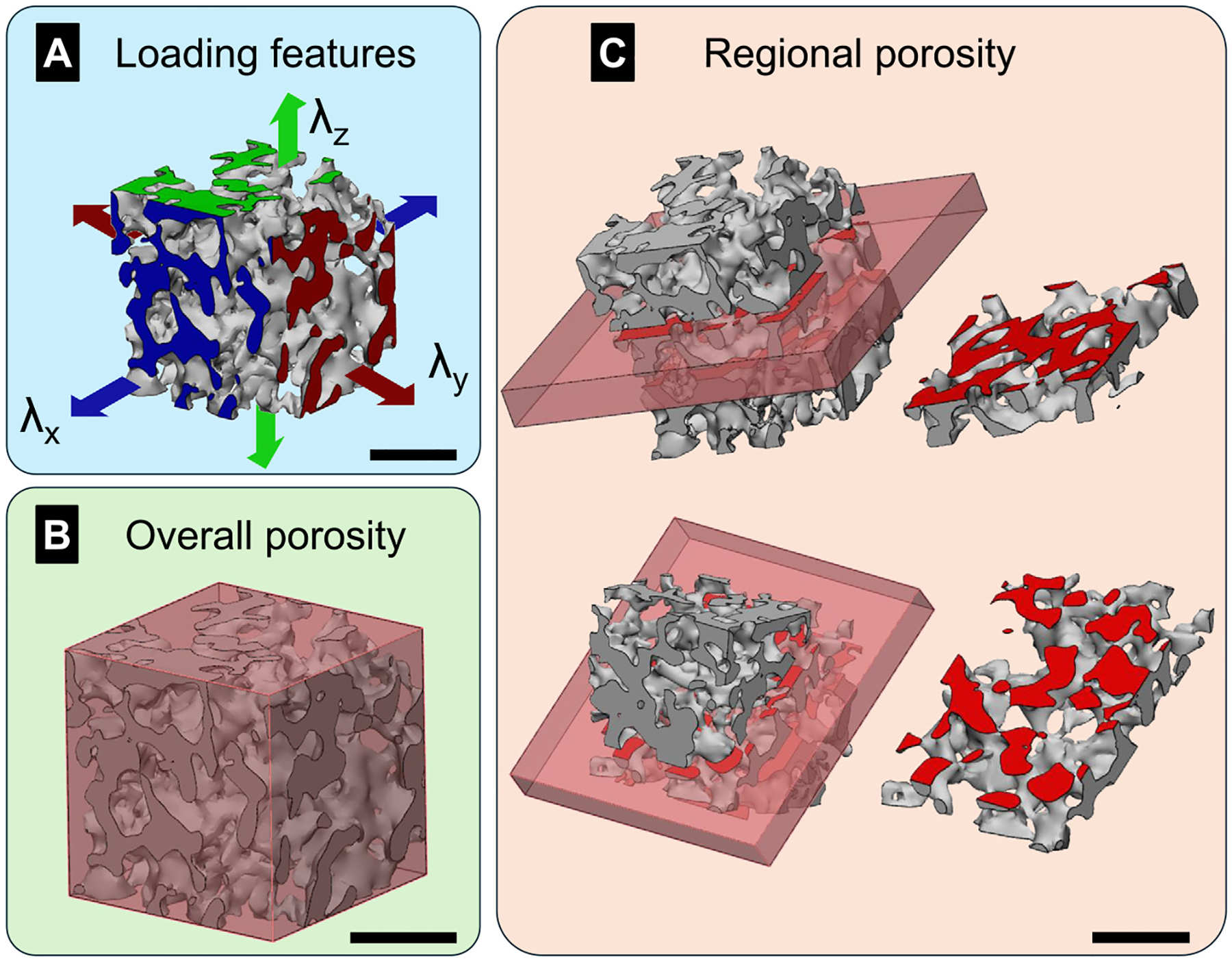
(**A**) Stretch boundary conditions applied on a representative tissue element (RTE). (**B**) Volumetric porosity of an RTE element. (**C**) Regional porosity of the cube, obtained by sectioning the RTE. Scale bars represent 1 mm.

The parenchymal tissue was modeled as an incompressible neo-Hookean material given by the strain energy function (Ψ)

(4) $$\Psi =\frac{\mu }{2}\left(I_{1}-3\right),$$

where μ is the shear modulus of the parenchymal tissue and $I_{1}$ is the first invariant of $\bar{\bar{C}}$ (trace, i.e., $I_{1}=\text{tr}(\bar{\bar{C}})$). For the current study, the shear modulus μ was chosen based on the values observed in the literature [20,22,31]. For the in-silico FE simulations, the RTE cubes face normal vectors aligned with the X, Y, and Z axes. The RTE cube faces were set to stretch by $\lambda_{x},\lambda_{y},\lambda_{z}$ along the three normal directions (Fig. 3A). For example, the faces with normals along the positive and negative x-axis were each stretched by an equal and opposite displacement such that the net stretch was $\lambda_{x}$. A single node point near the center of the mesh was held fixed. All the FE simulations were carried out in ABAQUS. The deformation protocols for the FE simulations were chosen to resemble the loading along the principal axes for a region of interest in the lung parenchyma. A complex deformation behavior represented by $\bar{\bar{F}}$ with all non-zero values can be reduced to a diagonal matrix represented by three principal stretches (eigenvalues). Thus, we subjected the RTEs to stretching by different values along all three directions. The limits of the stretch values were determined by the mesoscale volumetric deformation (J) observed in our previous study [17]. To obtain 0.5<J<3.0, the limits of the individual stretch values were set to 0.8<λ<1.5. Eq. (3) was used to estimate $\bar{\bar{C}}$ whose eigenvalues identify $\lambda_{1},\lambda_{2}$ and $\lambda_{3}$.

Each RTE was tested using a unique set of stretch values, yielding 300 data points. The set of 3 stretch values for simulations was determined by Latin hypercube sampling (LHS). LHS was used to generate an even distribution of points in the sample space and to avoid clustering of stretch values. Stretching along all three directions was included to ensure that the artifical neural network (ANN) model was trained for all manners of deformation. While alveolar interdependence can prevent certain deformations in the training space from occurring during physiological respiration [32,33], training the ANN model over a comprehensive range will ensure that it can accurately predict stress from any strain tensor estimated via image registration. The maximum, minimum, and average principal stresses were obtained from each simulation to train the machine learning model.

### 2.3. Feature engineering of the RTEs

Twenty-five features were used in the input layer to train the machine learning model. The features consisted of material stiffness, three stretch values (Fig. 3A), volumetric porosity (Fig. 3B), and 21 regional porosity values. 15 of the 21 regional porosity values were obtained by sectioning the RTE along the X, Y, and Z axes (Figs. 3C), with a section thickness of 0.4 mm. The remaining six regional porosity values were values obtained by sectioning the RTE along its face diagonals (Figs. 3C), with a section thickness of 0.56 mm. We condensed the stress matrix into normal (hydrostatic) and shear stresses for the output layer. For each element in the RTE, the stress was given by

(5) $$\bar{\bar{\sigma }}=\left[\begin{array}{lll} \sigma_{xx} & \sigma_{xy} & \sigma_{xz}\\ \sigma_{yx} & \sigma_{yy} & \sigma_{yz}\\ \sigma_{zx} & \sigma_{zy} & \sigma_{zz} \end{array}\right].$$

The eigenvalues (principal) were estimated, denoted by $\sigma_{1},\sigma_{2}$, and $\sigma_{3}$ (where $\sigma_{1}\geq \sigma_{2}\geq \sigma_{3}$). Using the principal stresses, the normal $\left(\sigma_{h}\right)$ and shear stresses $\left(\tau_{\text{max}}\right)$ were estimated through the equations,

(6) $$\sigma_{h}=\frac{\sigma_{1}+\sigma_{2}+\sigma_{3}}{3}=\frac{\sigma_{xx}+\sigma_{yy}+\sigma_{zz}}{3},\text{and}$$

(7) $$\tau_{\text{max}}=\left|\frac{\sigma_{1}-\sigma_{3}}{2}\right|,$$

respectively.

### 2.4. RTE geometry augmentation and machine learning

**Fig. 4.**
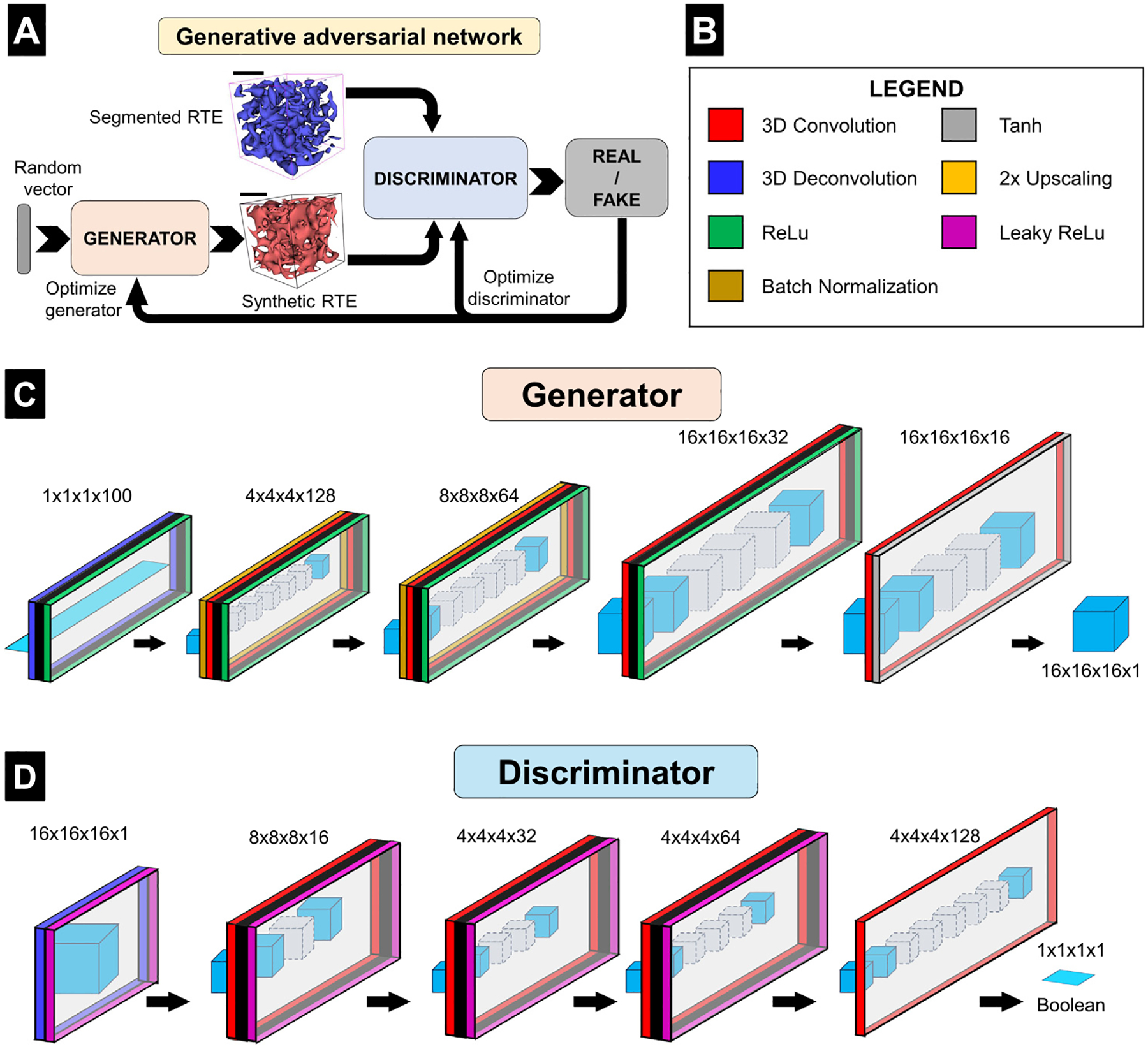
(**A**) Schematic of the method used to train the generative adversarial network (GAN). (**B**) Legend of the components used in the generator and discriminator. Schematic of the (**C**) generator and (**D**) discriminator used. Scale bar in (**A**) represents 1 mm.

**Fig. 5.**
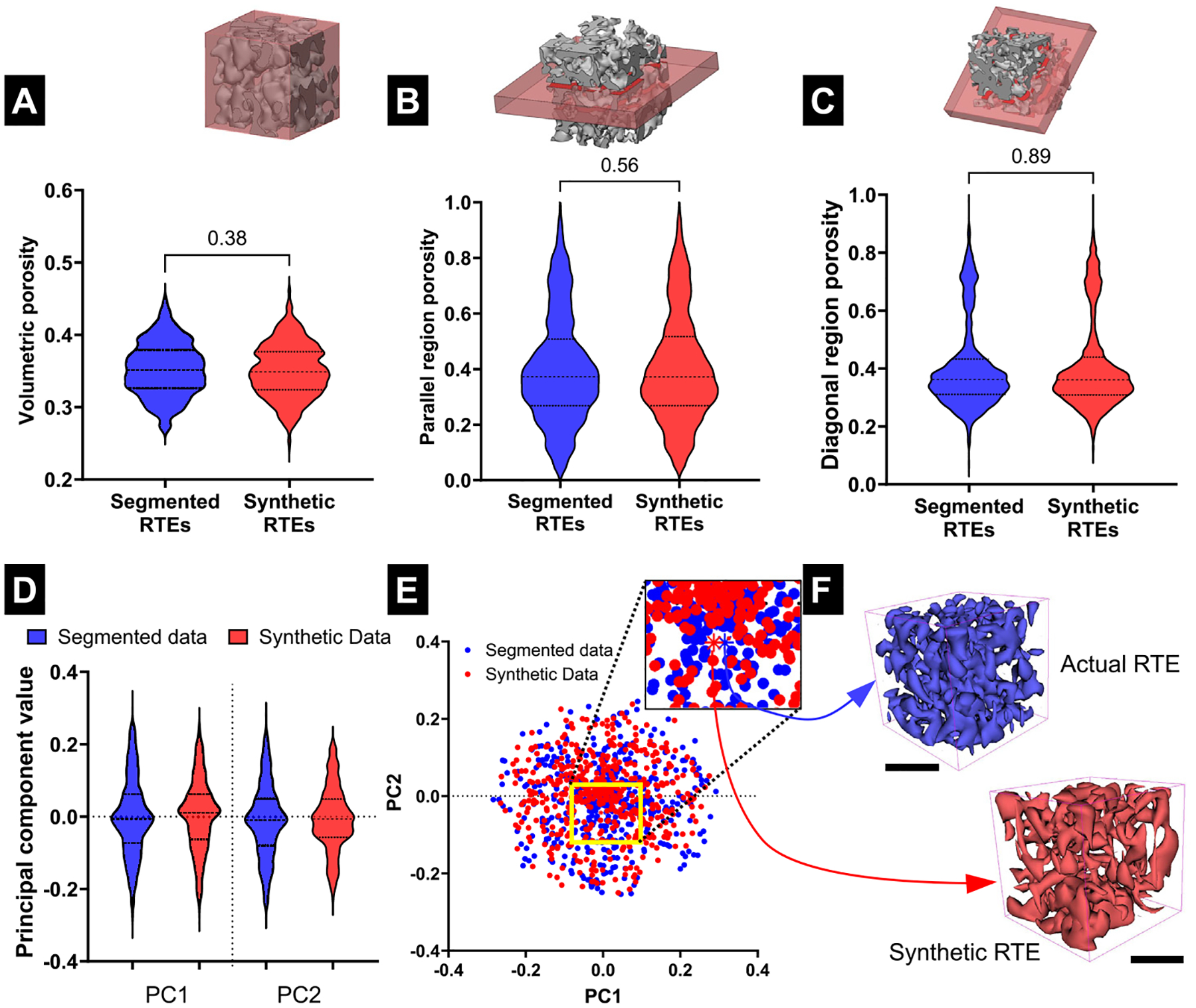
Geometric features of the segmented and synthetic representative tissue elements (RTEs): (**A**) volumetric porosity, (**B**) regional porosity at the parallel plane sections, and (**C**) regional porosity at the diagonal plane sections. Representative visualization is presented in the inset figures in (**A–C**). (**D**) The first two principal components (PC), (**E**) PC1 and PC2, plotted against each other for segmented (n=300) and synthetically generated RTEs (n=750), (**F**) visualization of representative RTEs from each group. Statistical analysis in (**A–C**) was performed using Student’s t-test. Scale bar in (**F**) represents 1 mm.

To enhance the training data to be used for the ML model, we used a GAN model to generate synthetic RTE geometries based on segmented RTE cubes obtained from X-ray microtomography (Fig. 4A). The GAN model consisted of a generator to produce synthetic RTE geometries from random noise (Figs. 4B, C) and a discriminator to decide if an RTE is “realistic” (Figs. 4B, D). The real and synthetic RTE geometries were passed through the discriminator, and the weights in both the generator and the discriminator were updated based on these results. The components were based on a study by Kwon et al. [34] and modified to work with a 3D array of size 16 × 16 × 16. To determine the quality of the RTEs generated by the GAN generator, we compared the geometric features of the synthetic RTEs to the segmented RTEs (Figs. 5A–C). In addition, we performed principal component analysis (PCA) on 500 synthetic RTEs and compared the PCA results with those from the 300 segmented RTEs in the original training data (Figs. 5D–F). The first two principal components were chosen and plotted for both the segmented and synthetic RTEs. After PCA analysis, 750 synthetically generated RTEs were used for in-silico simulations. Thus, a total of 1050 RTEs were subjected to FE simulations, with 300 of those being reconstructed from μCT images.

For the machine learning model, the input features are the strains and geometric features, and the output features are the stresses obtained from the FE simulations. Since there is a non-linear relationship between the strains and stresses, we used a multi-layer perceptron (MLP) for this work. The model consisted of an input layer with 25 units, four hidden layers with 512 units each, and an output layer with two units (Fig. S1) for the average normal and shear stresses in the RTE. The MLP training was performed twice, once with just the RTEs obtained through segmentation and reconstruction and once with both the segmented and synthetic RTEs.

The ANN model used the Adaptive Moment Estimation (Adam) optimizer. The model was trained for 500 epochs with the loss function set to mean squared error (MSE) and the learning rate set to 0.0002. For the initial training, the data was split into 150, 50, and 100 points for training, validation, and testing, respectively. When the synthetic RTEs were included in the MLP training, 500 synthetic RTEs were incorporated into the training data for a total of 650 training points. 250 synthetic RTEs were incorporated into the validation data, resulting in a total of 300 validation points. The test data remained constant between both rounds of MLP training, and was kept separated from the training and validation sets. Thus, there was no data leakage between the training, validation, and test datasets. A repetition of the training runs would be tested on a different set of 100 segmented RTEs. Thus, we ensured the model was not overtrained by keeping the testing dataset constant. In addition, the training with the augmented synthetic RTEs was repeated, with a weighted loss function that was biased towards segmented RTEs. The bias was set to a 4:1 ratio when estimating the squared error.

### 2.5. Application to radiation-induced lung injury

After training the ML model, we applied it to assess longitudinal alterations in SWS in a rat model of radiation-induced lung injury (RILI). The animal model used in this study was based on the model reported by Loza et al. [35]. Approval for procedures involving animal subjects was obtained from the University of Pennsylvania IACUC. Briefly, one male Sprague-Dawley (SD) rat (n=1) underwent right lung irradiation of 20 Gy in a single fraction using a small-animal radiotherapy device (SARRP, Xstrahl) and was returned to housing for 5 months to allow RILI to develop. The rat was imaged prior to radiation using dynamic computed tomography (4DCT) under isoflurane anesthesia in a free-breathing state, capturing 16 image stacks per respiratory cycle. 4DCT scans were then acquired longitudinally at the 1-month, 3-month, and 5-month post-radiation timepoints. Lung volumes were approximated by estimating the number of voxels within the lung whose Hounsfield unit values were below −800. To assess the kinematic behavior, we used the dynamic imaging and image registration method detailed in our previous study [17]. Briefly, an open-source image registration code (Nifty-reg) was used to perform non-rigid image registration to obtain voxel-based displacement during respiration and, subsequently, compute the deformation. In these images, the end-expiration timepoint was used as a reference image, and the end-inspiration timepoint was used as the deformed image whose strain was to be calculated. The lungs were segmented from the reference image stack using 3DSlicer, and the segmentation was propagated through image registration to track the region of interest throughout the inspiratory cycle. An additional two rats (n=2) were used for histological analysis. One rat was subject to the same 20 Gy single fraction radiation as the rat used for longitudinal imaging, while the other rat served as the control. Both rats were sacrificed and used for histological imaging 6 weeks post radiation.

The voxel-wise displacement from the end-expiratory timepoint to the end-inspiratory timepoint was used to estimate the deformation gradient $\bar{\bar{F}}$ and the Green-Lagrange strain $\bar{\bar{E}}$. The eigenvalues of $\bar{\bar{E}}$ (principal strains) were estimated, denoted by $E_{1},E_{2}$ and $E_{3}$ (where $E_{1}\geq E_{2}\geq E_{3}$). Using the deformation gradient and principal strains, the volumetric strain (J) and the shear strain $\left(\gamma_{\text{max}}\right)$ were estimated through the equations,

(8) $$J=\text{det}(\bar{\bar{F}})=\sqrt{\text{det}(\bar{\bar{C}})},$$

(9) $$\begin{array}{c} \gamma_{max}=\left|\frac{E_{1}-E_{3}}{2}\right|, \end{array}$$

respectively. In addition to the strains obtained through the respiratory cycle, an average organ-level compliance was obtained by assuming an equal pressure difference (atmospheric pressure minus alveolar pressure) and estimating the change in overall lung volume. The compliance was assumed to be constant throughout the lungs. This organ-level compliance was used to estimate an organ-level shear modulus (μ). The geometric features were estimated for each image of the 4DCT stack. An RTE cube of side 2 mm was assumed around each voxel, and the volumetric porosity was estimated from the average Hounsfield unit values (voxel intensity) for the RTE. The regional porosity features were estimated by isolating appropriate voxels from the RTE and forming planes. The strains, estimated as the average strain in the RTE, were combined with stiffness and geometric features and used as inputs to the trained ML model to estimate the maximum and average principal stresses in each voxel. The stresses and strains were analyzed to understand the evolution of RILI on lung function.

### 2.6. Statistical analysis

**Fig. 6.**
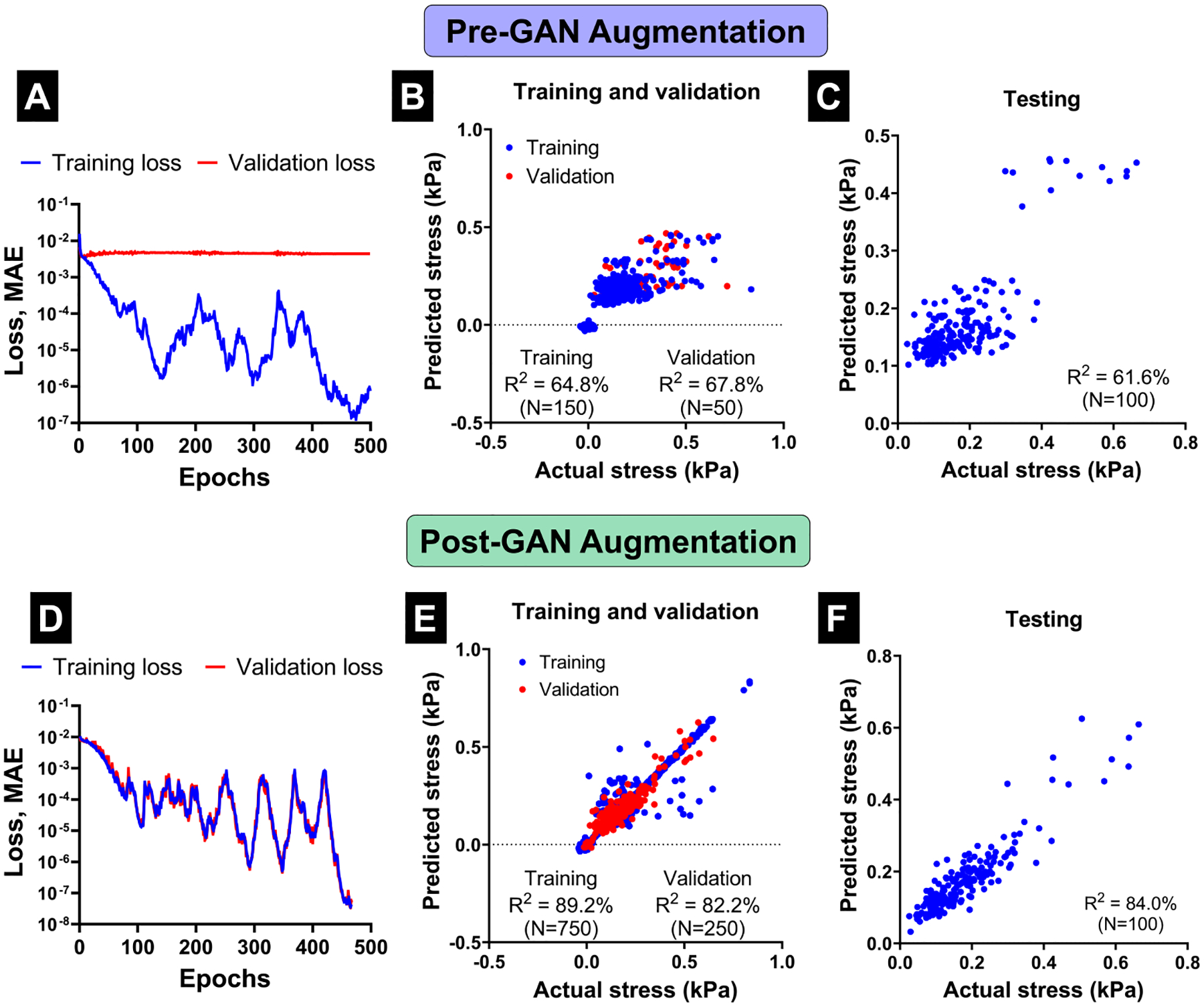
(**A**) Training and validation loss during training of segmented representative tissue elements (RTEs). (**B**) Training and validation accuracy during the training of segmented RTEs. (**C**) Testing accuracy during training of segmented RTEs. (**D**) Training and validation loss during training of segmented and synthetic RTEs. (**E**) training and validation accuracy during the training of segmented and synthetic RTEs. (**F**) Testing accuracy during training of segmented and synthetic RTEs. MAE — mean absolute error.

**Fig. 7.**
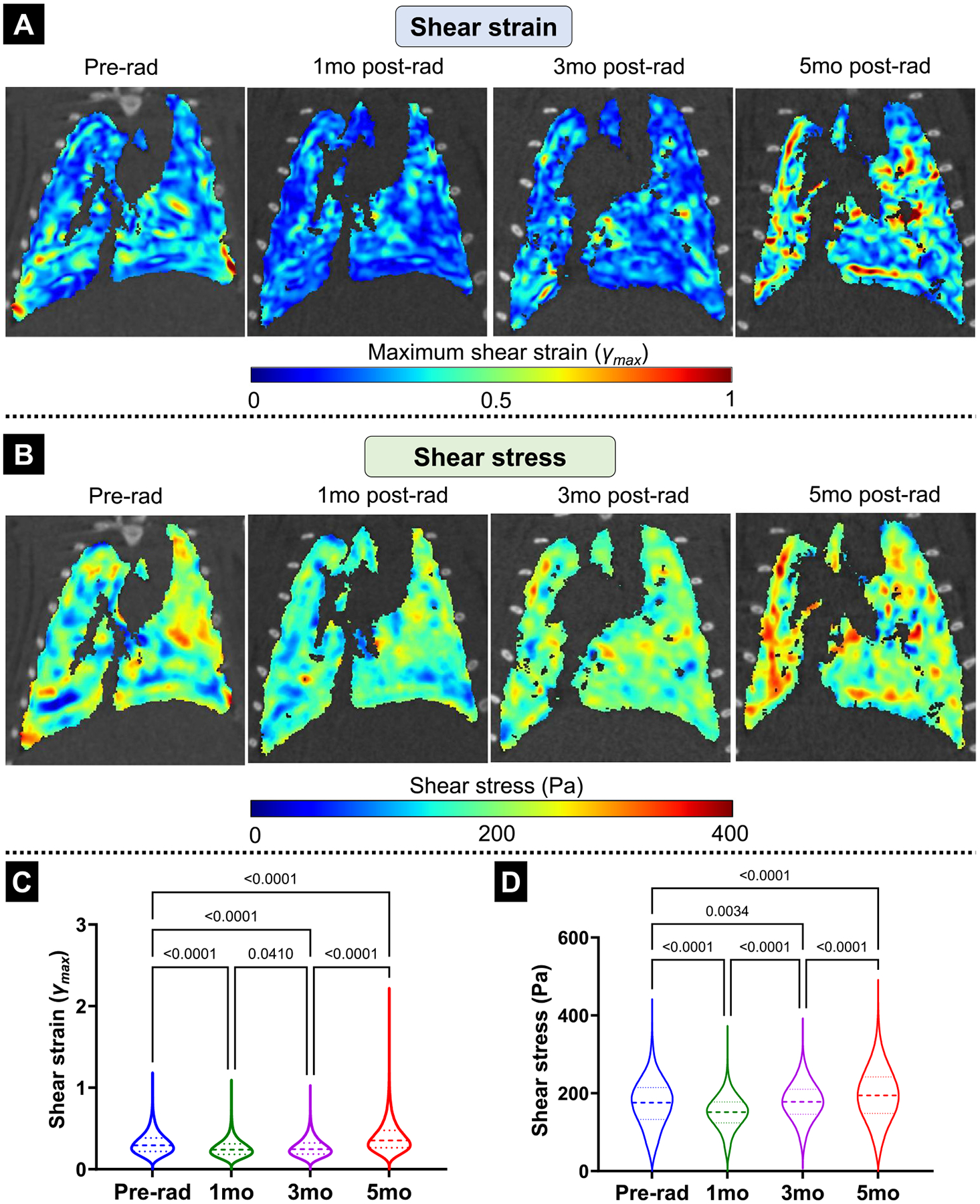
(**A**) Shear strain $\left(\gamma_{max}\right)$ estimated at the pre-rad, 1-month (1mo) post-rad, 3-month (3mo) post-rad, and 5-month (5mo) post-rad timepoints. (**B**) Shear stress $\left(\tau_{max}\right)$ estimated at the pre-rad, 1mo post-rad, 3mo post-rad, and 5mo post-rad timepoints. Violin plots of (**C**) shear strain and (**D**) shear stress distribution. Statistical analysis in (**C**) and (**D**) was performed using one-way ANOVA with Tukey’s correction for multiple comparisons. pre-rad: pre-radiation, mo post-rad: months post-radiation.

The experimental data were analyzed in Microsoft Excel and GraphPad Prism 9. All the data are represented as mean ± standard deviation. The data presented in Figs. 5A, B, and C were analyzed using unpaired Student t tests. The data presented in Figs. 6B, C, E, and F were analyzed using linear regression, with the square of the Pearson correlation being reported as the goodness of fit. The data presented in Figs. 7 and S4 were analyzed using one-way ANOVA with Tukey’s correction when performing statistical analysis with a dependent variable. Significance was accepted at p<0.05. For this study, we have chosen to report the numerical significance values [36]. In addition to performing one-way ANOVA comparisons between the stresses and strains at the various timepoints for the RILI models (Figs. 7 and S7), the probability distributions were compared through relative entropy, computed using the Jensen–Shannon divergence (JSD), via the following equations

(10) $$\text{JSD}(P\|Q)=\frac{1}{2}D(P\|M)+\frac{1}{2}D(Q\left\|M\right),\;M=\frac{1}{2}\left(P+Q\right),$$

(11) $$D(P\|M)=\sum_{x\in \chi }P\left(x\right){\text{log}}_{10}\left(\frac{P\left(x\right)}{M\left(x\right)}\right).$$

Here, P and Q are two probability distribution functions defined on a sample space χ⋅D(P‖M) defines the Kullback–Leibler divergence. The JSD was chosen because it is a symmetric function (JSD(P‖Q)=JSD(Q‖P)) and its values are always finite. It should be noted that 0 ≤ JSD ≤ 0.301, with zero indicating identical distributions and 0.301 indicating no overlap between the distributions. The relative entropy between the timepoints has been reported in the supplementary material (Fig. S8).

## 3. Results

### 3.1. FE simulation of RTEs indicated significant stress concentration

FE simulations of RTEs indicated that the maximum stress occurred in thinner regions and other regions prone to stress concentration (Fig. S2A). The ratio of the maximum principal stress (Table 1) to the average normal stress (Table 1) was 3 ± 5.4, indicating a notable amount of stress concentration. In addition, negative principal stresses were observed, suggesting that regions in the RTE undergo compression despite the RTE being stretched (Fig. S2B).

### 3.2. Synthetic RTEs demonstrated similar properties to those of segmented RTEs

Prior to comparing the geometric features and principal components of the segmented and synthetic RTEs, we compared these features between the RTEs segmented from the three rats to ensure minimal variation in parenchymal microstructure between different animal specimens. The results indicated that differences in geometric features and principal components were not statistically significant (Fig. S3). Although there were variations in the distribution of volumetric porosity between the three animals, the overall difference was not statistically significant. Comparing the geometric features of the synthetic RTEs generated by GAN with the segmented RTEs indicated that the volumetric porosity values were similar (n=300 for the segmented RTEs and n=750 for the synthetic RTEs; Fig. 5A). In addition, the regional porosity in the parallel plane regions (Fig. 5B) and in the diagonal plane regions (Fig. 5C) were similar between the two groups. Statistical significance between the segmented and synthetic RTEs was estimated for three groups of geometric features using unpaired Student’s t-tests. The results indicated that the difference between the segmented RTE and synthetic RTEs was not significant in all three geometric features (Figs. 5A–C). After performing a PCA analysis on the synthetic RTEs generated by the GAN model, the first principal component was −0.5 × 10^−3^ ± 0.1 for the actual data and 2.7 × 10^−3^ ± 0.07 for the synthetic data (Fig. 5D). The second principal component was −1.1 × 10^−2^ ± 0.1 for the actual data and −3.5 × 10^−3^ ± 0.1 for the synthetic data (Fig. 5D). When the first two principal components were plotted against each other, the actual and synthetic data occupied the same region (Fig. 5E), indicating that the GAN model is able to produce RTEs that are quantitatively similar to the actual RTEs obtained from segmentation (Fig. 5F). In addition, we compared the higher-order principal components to ensure that the GAN model captured small variance contributions (Table S1). The difference between corresponding higher-order principal components of the segmented and synthetic RTEs was statistically insignificant (Table S1). After the simulation of the synthetic RTEs, the stress distributions between the segmented and synthetic RTEs were compared, and results indicated similar distributions whose differences were not statistically significant (Fig. S4).

### 3.3. Accuracy of MLP model was enhanced with the incorporation of synthetic RTEs

When training the MLP with only segmented RTEs, the loss function plateaued around 200 epochs with an MSE of 3.2 × 10^−7^) and 0.013 for the training and validation loss, respectively (Fig. 6A). While training loss decreased with epochs, validation loss increased, indicating insufficient data. The model’s training and validation accuracies were 64.8% and 67.8%, respectively (Fig. 6B). The test accuracy was 61.6% (Fig. 6C) with the fixed testing data. When training the MLP with the data from both synthetic and segmented RTEs, the loss function plateaued around 100 epochs with an MSE of 1.1 × 10^−6^ and 8.7 × 10^−6^ for the training and validation loss, respectively (Fig. 6D). In this case, the training and validation loss decreased with the number of epochs, indicating sufficient data for training. The training and validation accuracy values increased to 89.2% and 82.2%, respectively (Fig. 6E). The testing accuracy when incorporating the synthetic RTEs also increased to 84.0% (Fig. 6F). When the loss function was weighted to favor segmented RTEs over synthetic RTEs (Fig. S5A), the training and validation accuracy decreased to 85.9% and 81.7%, respectively (Fig. S5B). The testing accuracy was between the original and augmented testing accuracy at 72.3% (Fig. S5C).

### 3.4. Shear stress increased in vivo in the rodent model of RILI post radiation

The ratio of end-inspiratory $\left(V_{EI}\right)$ to end-expiratory $\left(V_{EE}\right)$ lung volumes increased for the 1-month post-radiation timepoint and then decreased up to the 5-month post-radiation timepoints so that $(V_{EI}-\left.V_{EE}\right)/V_{EE}$ at 1-month post-radiation = 0.49, 2-month post-radiation = 0.51, 3-month post-radiation = 0.42, and at 5-month post-radiation = 0.40. In comparison, this ratio at pre-radiation was 0.45. Since the animal was free breathing (not mechanically ventilated), this indicates a slight increase in lung compliance up to 1-month post-radiation, followed by stiffening of the lungs. Histological analysis of rats at 6 weeks post-radiation indicated that the alveolar space in the irradiated lung featured moderate focal histiocytosis and mild multifocal alveolar edema (Fig. S6). In addition, the alveolar septa displayed moderate multifocal lymphohistiocytic interstitial inflammation. The histological slices also indicated that 40% of the pulmonary tissue was affected in the irradiated lung, as compared to 5% in the non-irradiated lung, indicating that the non-irradiated lung was not completely shielded from the radiation.

When investigating the lung strains, we observed increased heterogeneity in the representative slices of both the volumetric (J) and shear strains $\left(\gamma_{\text{max}}\right)$ at the 5-month post-radiation timepoint (Figs. 7A, S7A). This increased strain heterogeneity was also observed in the representative stress contours (Figs. 7B, S7B), and was more prominent in the normal stress $\left(\sigma_{h}\right)$ as compared to the shear stress $\left(\tau_{\text{max}}\right)$. When investigating the distribution of stress and strain, we observed a decrease in $\gamma_{\text{max}}$ up to the 3-month post-radiation timepoint (Fig. 7C), followed by a significant increase at the 5-month post-radiation timepoint (Table. S2). The volumetric strain was divided into two regions: the expanding region (J>1) and the contracting region (J<1). Average J in the expanding region was reduced at the 1-month and 3-month post-radiation timepoints, before increasing at the 5-month post-radiation timepoint (Fig. S7C, Table S2). J in the contracting region behaved the opposite, with an increased average at the 1-month and 3-month post-radiation timepoints before decreasing at the 5-month post-radiation timepoint. In addition, the fraction of volume with J>1 remained constant until the 3-month post-radiation timepoint before increasing at the 5-month post-radiation timepoint (Table S2). This suggests the presence of injured regions (low J) and over-ventilated/stretched healthy regions (high J and $\gamma_{\text{max}}$). The mean $\sigma_{h}$ remained constant, but the maximum $\sigma_{h}$ decreased mildly at the 1-month post-radiation timepoint, then increased at the 5-month post-radiation timepoint. The maximum values at the 5-month post-radiation timepoint were greater than those observed pre-radiation. However, the distribution indicated a larger spread in $\sigma_{h}$ at the 5-month post-radiation timepoint as compared to the pre-radiation timepoint (Fig. S7D). The mean $\tau_{\text{max}}$ also remained constant, but the maximum $\tau_{\text{max}}$ decreased up to the 3-months post-radiation timepoint, followed by an increase at the 5-month post-radiation timepoint (Fig. 7D). The increased stress heterogeneity based on the representative contours suggests the presence of localized volutrauma due to parenchymal tethering.

When comparing the distribution of stresses and strains over different timepoints using relative entropy (Fig. S8), the non-zero values indicate that no two distributions are identical. In general, the strains indicate greater differences between distributions as compared to their corresponding stress values. In shear strain, the greatest difference between the distributions was between the pre-radiation and 5-month post-radiation timepoints (Fig. S8A). In contrast, the corresponding difference in the shear stresses was the smallest (Fig. S8B). When comparing volumetric strains, the greatest difference between distributions was observed between the pre-radiation and 3-month post-radiation timepoints (Fig. S8B). In contrast, the greatest difference in normal stresses was observed between the 3-month and 5-month post-radiation timepoints (Fig. S8D). The small overall difference between the distributions of the pre-radiation and 5-month post-radiation timepoints suggests that fibrosis may act as a compensatory mechanism to restore lung function after the degenerative effects of radiation.

## 4. Discussion

### 4.1. Parenchymal architecture is a key factor in the accurate estimation of regional stresses in the lungs

We observed a significant stress concentration in the FE simulation of the RTEs, due to the microstructure of the lung parenchyma. The maximum principal stress was significantly higher than the mean stress (Table 1; representative example presented in Fig. S2). In the training data used for the study, we observed that the maximum principal stresses were one to two orders of magnitude greater than the expected maximum stress, for a given value of stretch, stiffness, and porosity of the RTE. This is in agreement with prior numerical investigations [22, 37]. The minimum principal stress contour showed that the RTE also had regions under compressive stress, despite being macroscopically elongated along the three axes. The presence of compressive principal stress could indicate local shear stresses, which could serve as an additional biomarker to track the progression of lung injury. In addition, maximum stress occurred where the parenchymal tissue was thinner, or in regions prone to stress concentrations, such as regions undergoing shear or bending deformation. Such regions in the lungs will be susceptible to damage and could potentially serve as the origin of larger, subsequently injured regions. Hence, generating various physiologically realistic RTEs becomes crucial to accurately estimating SWS. In addition, lung parenchymal tissue is commonly modeled as a poroelastic material, when the parenchymal microstructure is not explicitly considered. In this study, the alveolar septal walls were modeled as incompressible neo-Hookean materials, and poroelasticity was captured through the geometry of RTEs. Since the RTEs are porous, an RTE cube effectively behaved as a non-linear poroelastic material.

### 4.2. Augmenting training data through synthetic RTEs substantially minimized the need for parenchymal microstructural imaging

The amplification of stress indicates the need to incorporate realistic geometries. However, the segmentation and reconstruction of RTEs are limiting factors when translating this method to human application. Towards this end, the GAN method presented in this study serves as an important tool for improving the quality of training data used in the MLP. The GAN model can generate a large number of “synthetic” RTEs, which can be filtered based on the expected porosity in healthy parenchymal tissue. In addition, GAN models can generate a large number of synthetic RTEs corresponding to pathological conditions, such as diseased and collapsed regions, from a small number of real RTEs obtained through reconstruction. Based on principal component analysis, we have demonstrated that the synthetic RTEs behave similarly to the RTEs obtained from segmentation. In addition, augmenting the training data with FE simulation results of the synthetic RTEs increases the accuracy of training, validation, and testing. When using the MLP to predict regional stress from in-vivo strain estimated through CT image registration, we observed that the maximum stress and stretch were significantly larger than their respective average in the presented section. This heterogeneity highlights the shortcomings of using organ-level averaged metrics.

### 4.3. Septal wall stress can reflect remodeling post radiation in RILI

After training the ANN model with synthetic RTEs to improve the accuracy, the model was used to investigate the evolution of SWS in a rodent model of RILI. One of the primary reasons for investigating the evolution of SWS in a rodent model of RILI was the ability to modulate severity by varying the radiation dose. Future studies will investigate the ML model’s ability to modulate injury severity by varying the number of doses and the radiation received by the rodents in each dose. The representative results investigating the effect of RILI suggested the presence of both volutrauma and atelectrauma, indicated by the heterogeneous volumetric strain and normal stress contours. The heterogeneities in the volumetric strain contour indicate the presence of underventilated or atelectatic regions (low volumetric strain) and over-ventilated, potentially healthy regions (high volumetric strain). We attribute this heterogeneous ventilation to increased stiffness or collapse of the injured regions, which leads to underventilation. Underventilation of the injured regions results in overventilation of adjacent healthy regions due to parenchymal tethering, potentially worsening injury.

The decrease in lung compliance (tissue stiffening) is expected due to the pathophysiological processes associated with radiation injuries [29]. The early phase, which occurs up to 3 weeks post-radiation, is characterized by increased lung compliance due to degenerative changes in the alveolar walls [38]. Following the early phase is the radiation pneumonitis phase, which occurs between 3 and 12 weeks post-radiation. Alveolar collapse is expected in this phase, driven by detachment of the alveolar endothelium and epithelium, alveolocapillary barrier disruption, and surfactant inactivation. The alveolar SWS is expected to remain constant in this phase in the absence of alveolar interdependence, as alveolar collapse and reopening do not affect alveolar deformation at larger expansions (i.e., near end-inspiration). However, the alveolar interdependence may affect stresses and strains if collapsed regions persist at higher inflation levels [33], a hypothesis that remains to be verified through further studies. The final phase is the fibrotic phase, occurring around 5–6 months post-radiation, where there is extensive deposition of collagen in the alveoli and pulmonary interstitium [39]. These fibrotic events are expected to reduce pulmonary volume and significantly decrease lung compliance. However, SWS is expected to be significantly higher in the fibrotic lung at comparable volume levels, owing to increased parenchymal tissue stiffness resulting from collagen fibrosis. The consistency between the trend in SWS and the pathophysiological processes associated with RILI serves to highlight the clinical applicability of such an ANN-based framework. While data for reconstructing parenchymal microstructure in clinics will primarily be limited to histological imaging from biopsy samples, the GAN-based method for augmenting data with synthetic RTEs should substantially enhance the translational potential for clinics. In addition, similar pathophysiological processes have been observed in ARDS, VILI, and acute lung injury. The framework proposed in this study could be extended to other types of acute lung injury.

### 4.4. Clinical translation and future work

Lung injuries, such as RILI and VILI, can cause significant heterogeneity in ventilation, leading to injury in healthy regions and worsening injury in injured regions. This can be exacerbated by mechanical ventilation via VILI mechanisms. In addition, lung injury is frequently accompanied by atelectasis, which can lead to stress concentrations that increase the occurrence and spread of injury [40]. The combination of SWS and kinematic biomarkers, such as strain, can improve understanding of the progression of lung injury. Future work will focus on incorporating surfactant biophysics and integrating diseased regions with surfactant-flooded and collapsed areas to capture the evolution of strain and stress in diseases such as RILI, ARDS, and VILI, where stress concentrations play a crucial role in the onset and spread of injury.

The method presented in this study has the potential to be applied to optimize mechanical ventilation settings and guide clinical interventions. While the method reported in this study uses dynamic CT imaging to estimate strain and then stress, it can also be extended to work on static CT imaging through our previous work [41,42]. In that study, we developed a method to estimate strains from static CT images acquired at the end-expiratory and end-inspiratory timepoints [41,42]. In addition, we developed a method to generate synthetic dynamic CT images from FE simulations [42,43], enabling conversion of CT images into dynamic CT images to assess the accuracy of the SWS biomarker using minimal 4DCT images. We expect the SWS biomarker to serve as a regional indicator of injury and thus improve subject-specific clinical intervention strategies. Future work will be focused on enhancing the translation potential of this method through the incorporation of physics-informed neural networks (PINN) to broaden the model’s learning of the relationship between lung strain and SWS. As parenchymal architecture plays an important role in this relationship, we expect a framework that incorporates both GANs and PINNs to reduce the need for a large number of segmented RTEs. In addition, future work will also incorporate RTEs from diseased regions to enhance the accurate prediction of SWS in injured regions and thus be better suited to predict the evolution of lung health in different diseases. Future studies will also assess the diagnostic capability of the proposed framework through evaluating lung tissue density, organ-level compliance, and inflammatory cytokine biomarkers such as tumor necrosis factor (TNF)-α, interleukin (IL)-6, and IL-10 [44]. While it is not feasible to image inflammatory biomarkers with CT, MRI or positron emission tomography (PET) can be used to assess the in-vivo expression of such markers. The expression of inflammatory markers will be used to assess the diagnostic accuracy of the SWS biomarker proposed in this work.

### 4.5. Limitations

Our training data in this study did not incorporate variation in the parenchymal regional stiffness. In the case study using the RILI animal model, we estimated organ-level average stiffness from volume, assuming that the pressure at maximum inflation was atmospheric (i.e., no pressure difference relative to the external atmosphere). This method may cause large errors in the case of collapsed airways or alveoli, caused by surfactant dysfunction. In cases of diseases with regional alterations in parenchymal behavior, the model would require additional information from images to determine altered regional stiffness. Pulmonary surfactant contributes significantly to the stiffness of the parenchymal tissue during respiration [45–47]. However, we have not incorporated the effect of pulmonary surfactant in the FE simulations used to train the ML model. The effect of pulmonary surfactant was not required to assess the SWS, as we used displacement boundary conditions in the FE simulations to train the ML model. Since the alveolar septal walls and the pulmonary surfactant are stretched in parallel, the stretch experienced by both elements is the same, and the forces are additive. Hence, the parenchymal tissue element can be analyzed independently when the system input is displacement. Future studies will incorporate the effects of pulmonary surfactant, providing insights into diseases with surfactant dysfunction, such as VILI and acute lung injury. Another limitation with the mechanical model considered in this study was the lack of incorporating tissue viscoelasticity. Lung parenchymal tissue is known to be strongly viscoelastic [14,24], and the combination of tissue viscoelasticity and the effects of pulmonary surfactant results in distinct pressure–volume curves during inspiration and expiration. While errors arising from the model’s lack of tissue viscoelasticity can be minimized with dynamic CT imaging, which offers high temporal resolution, these effects must be accounted for when using static CT imaging to estimate lung strains. Future studies will investigate the effect of both pulmonary surfactant and parenchymal viscoelasticity on SWS and regional pressures.

The μCT imaging performed on fixed inflated lung tissue may have been at a higher pressure and volume than the physiological range. As summarized by Perlman et al. [48], inflation fixation removes the air–liquid interface, significantly contributing to lung stiffness and leading to inflation to supraphysiologic volumes. This would cause the alveolar structures to be in an expanded state when imaged, potentially leading to errors in FE simulations and ANN training. Future studies will explore the effect of inflation pressure during fixation on the reconstructed RTE geometry. In addition, intratracheal fixation can recruit atelectatic regions. Although collapsed alveoli are not commonly observed in healthy lungs during physiological respiration, they can become an important determinant of local parenchymal behavior in diseases such as ARDS and VILI. The incorporation of collapsed alveolar structures through imaging and reconstruction of diseased lung tissue, and the incorporation of alveolar unfolding/recruitment behavior, will be explored in future studies. A limitation regarding the synthetic RTEs was that all the segmented RTEs used for FE simulations and the subsequent training of the ANN model were used to train the GAN model. Thus, it would be expected that a portion of the synthetic RTEs would be geometrically similar to segmented RTEs in the test set for the ANN model. Since the stresses in the RTEs are strongly dependent on their architecture, synthetic RTEs similar to the segmented RTEs in the test set would improve the accuracy of the ANN model. Future studies will use an expanded cohort of rats for μCT imaging, ensuring that the RTEs in the testing group and those used to train the GAN are mutually exclusive.

## 5. Conclusion

In this study, we have presented a new regional biomarker, septal wall stress (SWS), to track lung health and a method to estimate SWS through a combination of dynamic imaging and machine learning. The machine learning model was trained using synthetic data from finite element (FE) simulations of representative tissue elements (RTEs) obtained from μCT imaging. In addition to the RTEs obtained through segmentation and reconstruction, we have presented a method to augment the number of RTE geometries using a GAN, thereby increasing the ANN model’s training and testing accuracy. In addition, when applied to the rodent RILI model, the ANN model captured the biomechanical behavior of pneumonitis and fibrosis associated with early- and late-stage remodeling post-radiation, respectively. We expect that regional stresses will provide key insights, including those obfuscated from global measures, into the onset and progression of lung injuries.

## Supplementary Material

1

## Figures and Tables

**Table 1:** Variation stress in the RTEs during FE simulations.

Parameter (n=300)	Value
mean normal stress (Pa)	221.8 ± 133.2
mean shear stress (Pa)	10.1 ± 9.4
maximum principal stress (Pa)	528.7 ± 245.8
minimum principal stress (Pa)	8.1 ± 55.4

## Data Availability

The data that support the findings of this study are available from the corresponding author, R.A., upon request.

## Statement of Significance

Injuries to the lung parenchymal tissue, such as radiation-induced lung injury (RILI), can lead to heterogeneous ventilation and reduced lung function. The drivers behind the onset and progression of such lung injuries are poorly understood. In this study, we developed a method to estimate alveolar septal wall stress (SWS) in vivo, through a combination of tissue mechanics, multiscale modeling, and machine learning (ML), to understand the effect of parenchymal biomechanics on the onset and progression of lung injuries. We applied the method to investigate a rodent model of RILI. The ML-based method could capture the pneumonitis and fibrosis behavior associated with early- and late-stage parenchymal remodeling post radiation, respectively. We expect that regional stresses in the lung parenchyma will provide key insights, including those obfuscated from global measures, into the onset and progression of lung injuries.

## Appendix A. Supplementary data

Supplementary material related to this article can be found online at https://doi.org/10.1016/j.actbio.2025.11.030.

## References

[1] Cereda M, Emami K, Xin Y, Kadlecek S, Kuzma NN, Mongkolwisetwara P, Profka H, Pickup S, Ishii M, Kavanagh BP, Deutschman CS, Rizi RR, Imaging the interaction of atelectasis and overdistension in surfactant-depleted lungs*, Crit. Care Med 41 (2) (2013). 10.1097/CCM.0b013e31826ab1f2 PMC3557664 23263577

[2] Cereda M, Xin Y, Hamedani H, Bellani G, Kadlecek S, Clapp J, Guerra L, Meeder N, Rajaei J, Tustison NJ, Gee JC, Kavanagh BP, Rizi RR, Tidal changes on CT and progression of ARDS, Thorax 72 (11) (2017) 981–989. 28634220 10.1136/thoraxjnl-2016-209833 PMC5738538

[3] Travis EL, Liao Z-X, Tucker SL, Spatial heterogeneity of the volume effect for radiation pneumonitis in mouse lung, Int. J. Radiat. Oncol. Biol. Phys 38 (5) (1997) 1045–1054. 9276371 10.1016/s0360-3016(97)00130-2

[4] Marks LB, Yu X, Vujaskovic Z, Small W, Folz R, Anscher MS, Radiation-induced lung injury, Semin. Radiat. Oncol 13 (3) (2003) 333–345. 12903021 10.1016/S1053-4296(03)00034-1

[5] Otto CM, Markstaller K, Kajikawa O, Karmrodt J, Syring RS, Pfeiffer B, Good VP, Frevert CW, Baumgardner JE, Spatial and temporal heterogeneity of ventilator-associated lung injury after surfactant depletion, J. Appl. Physiol 104 (5) (2008) 1485–1494. 18323462 10.1152/japplphysiol.01089.2007 PMC2459256

[6] Jain SV, Kollisch-Singule M, Satalin J, Searles Q, Dombert L, Abdel-Razek O, Yepuri N, Leonard A, Gruessner A, Andrews P, Fazal F, Meng Q, Wang G, Gatto LA, Habashi NM, Nieman GF, The role of high airway pressure and dynamic strain on ventilator-induced lung injury in a heterogeneous acute lung injury model, Intensiv. Care Med. Exp 5 (1) (2017). 10.1186/s40635-017-0138-1 PMC5427060 28497420

[7] Herrmann J, Kollisch-Singule M, Satalin J, Nieman GF, Kaczka DW, Assessment of heterogeneity in lung structure and function during mechanical ventilation: A review of methodologies, J. Eng. Sci. Med. Diagn. Ther 5 (4) (2022) 040801. 35832339 10.1115/1.4054386 PMC9132008

[8] Zompatori M, Ciccarese F, Fasano L, Overview of current lung imaging in acute respiratory distress syndrome, Eur. Respir. Rev 23 (134) (2014) 519–530. 25445951 10.1183/09059180.00001314 PMC9487404

[9] Suki B, Bartolák-Suki E, Biomechanics of the aging lung parenchyma, in: Mechanical Properties of Aging Soft Tissues, Springer International Publishing, Cham, 2015, pp. 95–133.

[10] Gattinoni L, Presenti A, Torresin A, Baglioni S, Rivolta M, Rossi F, Scarani F, Marcolin R, Cappelletti G, Adult respiratory distress syndrome profiles by computed tomography, J. Thorac. Imaging 1 (3) (1986) 25–30. 10.1097/00005382-198607000-00005 3298678

[11] Gattinoni L, Caironi P, Pelosi P, Goodman R, What has computed tomography taught us about the acute respiratory distress syndrome? Am. J. Respir. Crit. Care Med 164 (9) (2001) 1701–1711. 11719313 10.1164/ajrccm.164.9.2103121

[12] Fain S, Schiebler ML, McCormack DG, Parraga G, Imaging of lung function using hyperpolarized helium-3 magnetic resonance imaging: Review of current and emerging translational methods and applications, J. Magn. Reson. Imaging 32 (6) (2010) 1398–1408. 21105144 10.1002/jmri.22375 PMC3058806

[13] Washko GR, Hunninghake GM, Fernandez IE, Nishino M, Okajima Y, Yamashiro T, Ross JC, Estépar RSJ, Lynch DA, Brehm JM, Andriole KP, Diaz AA, Khorasani R, D’Aco K, Sciurba FC, Silverman EK, Hatabu H, Rosas IO, Lung volumes and emphysema in smokers with interstitial lung abnormalities, N. Engl. J. Med 364 (10) (2011) 897–906. 21388308 10.1056/NEJMoa1007285 PMC3074462

[14] Bel-Brunon A, Kehl S, Martin C, Uhlig S, Wall W, Numerical identification method for the non-linear viscoelastic compressible behavior of soft tissue using uniaxial tensile tests and image registration – application to rat lung parenchyma, J. Mech. Behav. Biomed. Mater 29 (2014) 360–374. 24184860 10.1016/j.jmbbm.2013.09.018

[15] Hurtado DE, Villarroel N, Retamal J, Bugedo G, Bruhn A, Improving the accuracy of registration-based biomechanical analysis: A finite element approach to lung regional strain quantification, IEEE Trans. Med. Imaging 35 (2) (2016) 580–588. 26441413 10.1109/TMI.2015.2483744

[16] Fu Y, Lei Y, Wang T, Higgins K, Bradley JD, Curran WJ, Liu T, Yang X, LungRegNet: An unsupervised deformable image registration method for 4D-CT lung, Med. Phys 47 (4) (2020) 1763–1774. 32017141 10.1002/mp.14065 PMC7165051

[17] Neelakantan S, Ismail MK, Mukherjee T, Smith BJ, Rizi R, Avazmohammadi R, Volumetric versus distortional deformation in rat lungs, in: Linte CA, Siewerdsen JH (Eds.), in: Medical Imaging 2023: Image-Guided Procedures, Robotic Interventions, and Modeling, vol. 12466, SPIE, International Society for Optics and Photonics, 2023, p. 124661R. 10.1117/12.2653648 PMC10414622 37565032

[18] Mariano CA, Sattari S, Maghsoudi-Ganjeh M, Tartibi M, Lo DD, Eskandari M, Novel mechanical strain characterization of ventilated ex vivo porcine and murine lung using digital image correlation, Front. Physiol 11 (2020) 1536. 10.3389/fphys.2020.600492 PMC7746832 33343395

[19] Nelson TM, Quiros KAM, Mariano CA, Sattari S, Ulu A, Dominguez EC, Nordgren TM, Eskandari M, Associating local strains to global pressure–volume mouse lung mechanics using digital image correlation, Physiol. Rep 10 (19) (2022) e15466, e15466 PHY2-2022-05-0219.R2. 36207795 10.14814/phy2.15466 PMC9547081

[20] Sarabia-Vallejos MA, Zuñiga M, Hurtado DE, The role of three-dimensionality and alveolar pressure in the distribution and amplification of alveolar stresses, Sci. Rep 9 (1) (2019) 8783. 31217511 10.1038/s41598-019-45343-4 PMC6584652

[21] Neelakantan S, Xin Y, Gaver DP, Cereda M, Rizi R, Smith BJ, Avazmohammadi R, Computational lung modelling in respiratory medicine, J. R. Soc. Interface 19 (191) (2022) 20220062. 35673857 10.1098/rsif.2022.0062 PMC9174712

[22] Concha F, Sarabia-Vallejos M, Hurtado DE, Micromechanical model of lung parenchyma hyperelasticity, J. Mech. Phys. Solids 112 (2018) 126–144.

[23] Concha F, Hurtado DE, Upscaling the poroelastic behavior of the lung parenchyma: A finite-deformation micromechanical model, J. Mech. Phys. Solids 145 (2020) 104147.

[24] Birzle AM, Wall WA, A viscoelastic nonlinear compressible material model of lung parenchyma – experiments and numerical identification, J. Mech. Behav. Biomed. Mater 94 (2019) 164–175. 30897504 10.1016/j.jmbbm.2019.02.024

[25] Berger L, Bordas R, Burrowes K, Grau V, Tavener S, Kay D, A poroelastic model coupled to a fluid network with applications in lung modelling, Int. J. Numer. Methods Biomed. Eng 32 (1) (2016). 10.1002/cnm.2731 26100614

[26] Xiao H, Xue X, Zhu M, Jiang X, Xia Q, Chen K, Li H, Long L, Peng K, Deep learning-based lung image registration: A review, Comput. Biol. Med 165 (2023) 107434. 37696177 10.1016/j.compbiomed.2023.107434

[27] Babaei H, Mendiola EA, Neelakantan S, Xiang Q, Vang A, Dixon RA, Shah DJ, Vanderslice P, Choudhary G, Avazmohammadi R, A machine learning model to estimate myocardial stiffness from EDPVR, Sci. Rep 12 (1) (2022) 5433. 35361836 10.1038/s41598-022-09128-6 PMC8971532

[28] Barahona J, Sahli Costabal F, Hurtado DE, Machine learning modeling of lung mechanics: Assessing the variability and propagation of uncertainty in respiratory-system compliance and airway resistance, Comput. Methods Programs Biomed 243 (2024) 107888. 37948910 10.1016/j.cmpb.2023.107888

[29] Arroyo-Hernández M, Maldonado F, Lozano-Ruiz F, Muñoz-Montaño W, Nuñez-Baez M, Arrieta O, Radiation-induced lung injury: current evidence, BMC Pulm. Med 21 (1) (2021) 9. 33407290 10.1186/s12890-020-01376-4 PMC7788688

[30] Luis AM, Radiotherapy for non-malignant diseases, Rep. Pr. Oncol. Radiother 18 (2013) S14–S15, XVII Congreso de la Sociedad Española de Oncologí a Radioterápica - XVII Meeting of the Radiation Oncology Spanish Society (SEOR).

[31] Rausch SMK, Haberthür D, Stampanoni M, Schittny JC, Wall WA, Local strain distribution in real three-dimensional alveolar geometries, Ann. Biomed. Eng 39 (11) (2011) 2835. 21607757 10.1007/s10439-011-0328-z

[32] Perlman CE, Lederer DJ, Bhattacharya J, Micromechanics of alveolar edema, Am. J. Respir. Cell. Mol. Biol 44 (1) (2011) 34–39. 20118224 10.1165/rcmb.2009-0005OC PMC3028256

[33] Knudsen L, Lopez-Rodriguez E, Berndt L, Steffen L, Ruppert C, Bates JHT, Ochs M, Smith BJ, Alveolar micromechanics in bleomycin-induced lung injury, Am. J. Respir. Cell. Mol. Biol 59 (6) (2018) 757–769. 30095988 10.1165/rcmb.2018-0044OC PMC6293074

[34] Kwon G, Han C, Kim D.-s., Generation of 3D brain MRI using auto-encoding generative adversarial networks, in: Shen D, Liu T, Peters TM, Staib LH, Essert C, Zhou S, Yap P-T, Khan A (Eds.), Medical Image Computing and Computer Assisted Intervention – MICCAI 2019, Springer International Publishing, Cham, 2019, pp. 118–126.

[35] Loza L, Ruppert K, Chen J, Hamedani H, Ismail M, Amzajerdian F, Duncan I, Kadlecek S, Rizi R, Assessing radiation-induced lung injury using a chemical shift imaging-based CSSR technique, in: B80–1. Methodological Advancements in Pulmonary Imaging, American Thoracic Society, 2024, A4494–A4494.

[36] Althouse A, Below J, Claggett B, Cox N, de Lemos J, Deo R, Duval S, Hachamovitch R, Kaul S, Keith S, Secemsky E, Teixeira-Pinto A, Roger V, Recommendations for statistical reporting in cardiovascular medicine: A special report from the American heart association, Circulation 144 (4) (2021) e70–e91. 34032474 10.1161/CIRCULATIONAHA.121.055393 PMC12850682

[37] Wall WA, Wiechert L, Comerford A, Rausch S, Towards a comprehensive computational model for the respiratory system, Int. J. Numer. Methods Biomed. Eng 26 (7) (2010) 807–827.

[38] Small W, Woloschak GE, Radiation toxicity: a practical medical guide, vol. 128, Springer Science & Business Media, 2006. 16335011

[39] Hanania AN, Mainwaring W, Ghebre YT, Hanania NA, Ludwig M, Radiation-induced lung injury: Assessment and management, Chest 156 (1) (2019) 150–162. 30998908 10.1016/j.chest.2019.03.033 PMC8097634

[40] Zimmermann R, Roeder F, Ruppert C, Smith BJ, Knudsen L, Low-volume ventilation of preinjured lungs degrades lung function via stress concentration and progressive alveolar collapse, Am. J. Physiol.-Lung Cell. Mol. Physiol 327 (1) (2024) L19–L39. 38712429 10.1152/ajplung.00323.2023

[41] Neelakantan S, Mukherjee T, Myers K, Rizi R, Avazmohammadi R, Physics-informed motion registration of lung parenchyma across static CT images, in: 2024 46th Annual International Conference of the IEEE Engineering in Medicine and Biology Society, EMBC, 2024, pp. 1–4. 10.1109/EMBC53108.2024.10781530 40039407

[42] Neelakantan S, Myers KJ, Avazmohammadi R, Physics-informed in-silico dynamic computed tomography of human lungs: Generation, evaluation, and refinement, J. Biomech. Eng 147 (10) (2025) 101008. 40802270 10.1115/1.4069391 PMC12617607

[43] Neelakantan S, Mukherjee T, Smith BJ, Myers K, Rizi R, Avazmohammadi R, In-silico CT lung phantom generated from finite-element mesh, in: Siewerdsen JH, Rettmann ME (Eds.), in: Medical Imaging 2024: Image-Guided Procedures, Robotic Interventions, and Modeling, vol. 12928, SPIE, International Society for Optics and Photonics, 2024, 1292829. 10.1117/12.3006973 PMC11270049 39055486

[44] Bhargava M, Wendt CH, Biomarkers in acute lung injury, Transl. Res 159 (4) (2012) 205–217. 22424425 10.1016/j.trsl.2012.01.007 PMC4537856

[45] Bachofen H, Schurch S, Urbinelli M, Weibel ER, Relations among alveolar surface tension, surface area, volume, and recoil pressure, J. Appl. Physiol 62 (5) (1987) 1878–1887. 3597262 10.1152/jappl.1987.62.5.1878

[46] Otis DR, Ingenito EP, Kamm RD, Johnson M, Dynamic surface tension of surfactant TA: experiments and theory, J. Appl. Physiol 77 (6) (1994) 2681–2688. 7896607 10.1152/jappl.1994.77.6.2681

[47] Ingenito EP, Mark L, Morris J, Espinosa FF, Kamm RD, Johnson M, Biophysical characterization and modeling of lung surfactant components, J. Appl. Physiol 86 (5) (1999) 1702–1714. 10233138 10.1152/jappl.1999.86.5.1702

[48] Perlman CE, Knudsen L, Smith BJ, The fix is not yet in: recommendation for fixation of lungs within physiological/pathophysiological volume range in preclinical pulmonary structure-function studies, Am. J. Physiol.-Lung Cell. Mol. Physiol 327 (2) (2024) L218–L231. 38712433 10.1152/ajplung.00341.2023 PMC11444500

