# Supplementary material for: Artificial intelligence-assisted multiscale lung modeling to predict alveolar septal wall stress
Source: Acta Biomater. Author manuscript; Available in PMC 2026 Jun 22. (PMC13284689; doi:10.1016/j.actbio.2025.11.030)
Supplement: 1 [file NIHMS2187319-supplement-1.pdf]

Supplementary Material

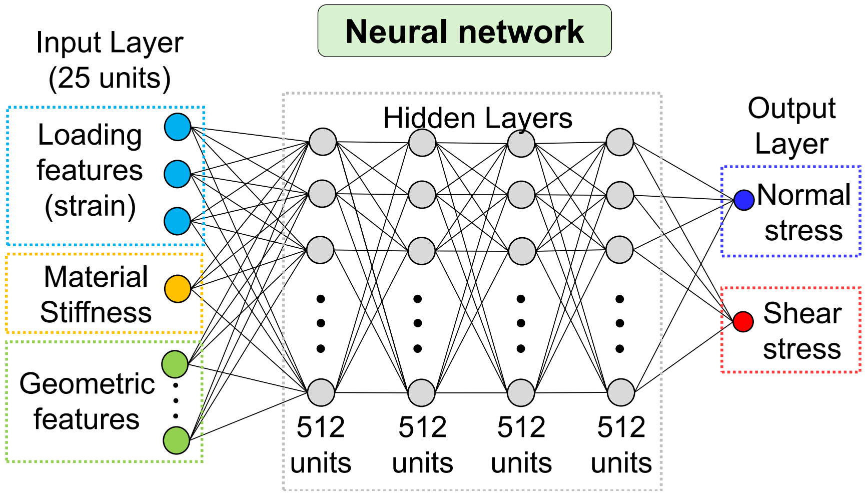

Figure S1. Schematic of the multi-layer perceptron (MLP) used to predict alveolar septal wall stress (SWS) in vivo.

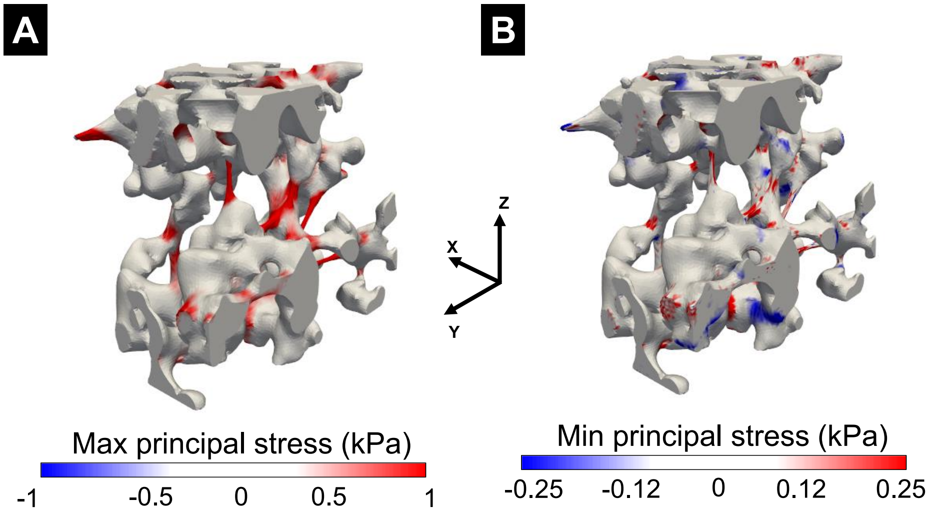

Figure S2. Representative in-silico simulation. (**A**) Maximum principal stress and (**B**) minimum principal stress. The applied stretch was $\lambda_x = 1.25$, $\lambda_y = 1.14$, $\lambda_z = 1.35$. Axis directions presented for reference.

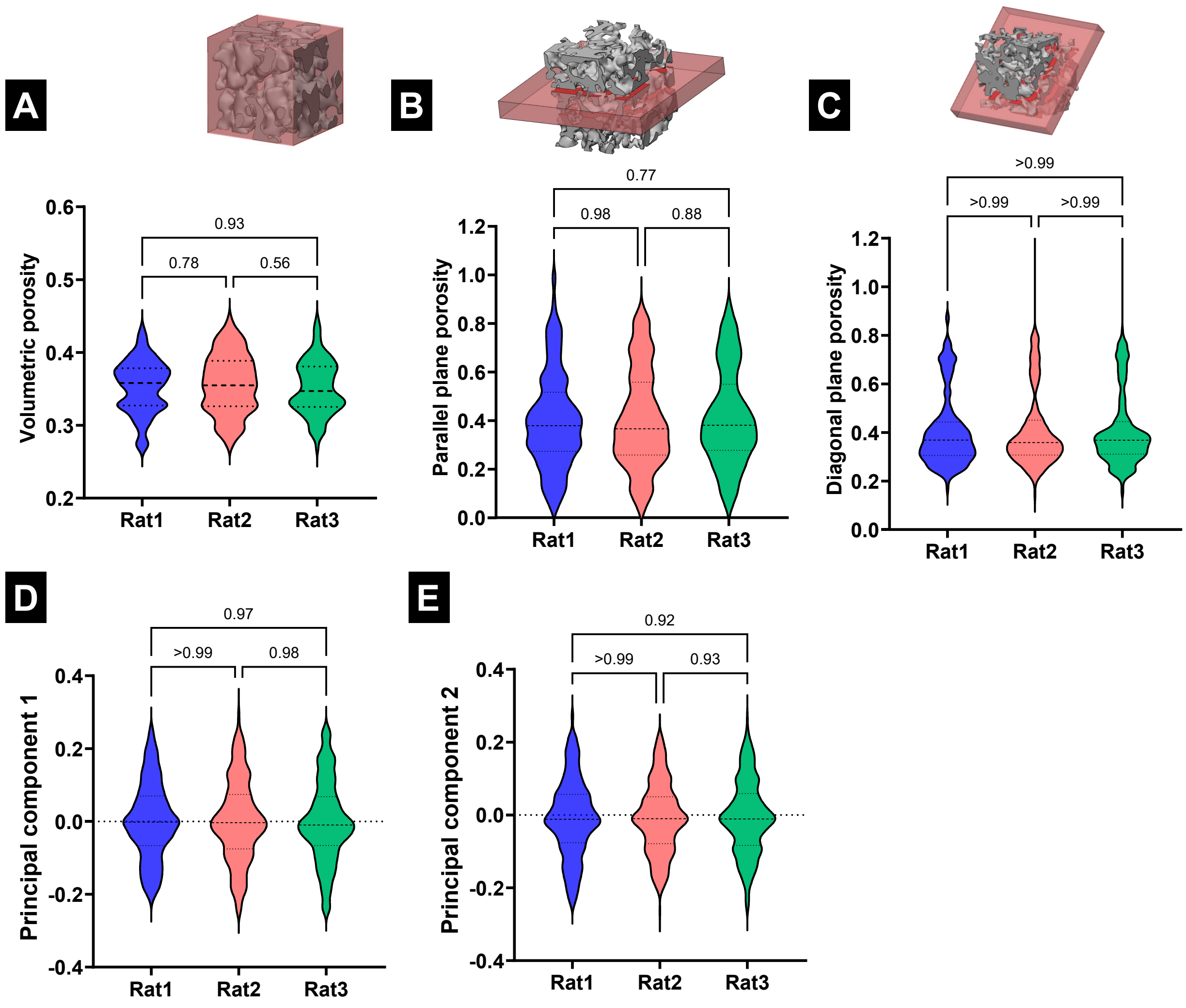

Figure S3. Comparison of the geometric features of the segmented representative tissue elements (RTEs) between the three rats: (**A**) volumetric porosity, (**B**) regional porosity at the parallel plane sections, and (**C**) regional porosity at the diagonal plane sections. Representative visualization is presented in the inset figures in (**A**-**C**). (**D**) The first principal component (PC) and (**E**) the second principal component. Statistical analysis was performed using one-way ANOVA with Tukey's correction for multiple comparisons.

Table S1. Variation higher order principal components. Statistical analysis was performed using Student's t-test.

Component	Segmented RTEs	Synthetic RTEs	p-value
3	$3.3 \times 10^{-3} \pm 4.2 \times 10^{-2}$	$4.1 \times 10^{-3} \pm 4.6 \times 10^{-2}$	0.770
4	$3.4 \times 10^{-3} \pm 3.8 \times 10^{-2}$	$7.1 \times 10^{-3} \pm 4.3 \times 10^{-2}$	0.148
5	$-3.4 \times 10^{-3} \pm 3.7 \times 10^{-2}$	$-1.7 \times 10^{-3} \pm 4.2 \times 10^{-2}$	0.499
6	$2.3 \times 10^{-3} \pm 3.6 \times 10^{-2}$	$5.8 \times 10^{-3} \pm 4.0 \times 10^{-2}$	0.149
7	$-1.1 \times 10^{-3} \pm 3.1 \times 10^{-2}$	$-1.8 \times 10^{-3} \pm 3.3 \times 10^{-2}$	0.742
8	$-1.1 \times 10^{-3} \pm 3.0 \times 10^{-2}$	$-2.9 \times 10^{-3} \pm 3.1 \times 10^{-2}$	0.350
9	$8.4 \times 10^{-4} \pm 3.0 \times 10^{-2}$	$-3.5 \times 10^{-5} \pm 3.1 \times 10^{-2}$	0.652
10	$1.9 \times 10^{-3} \pm 2.9 \times 10^{-2}$	$1.5 \times 10^{-3} \pm 3.0 \times 10^{-2}$	0.834

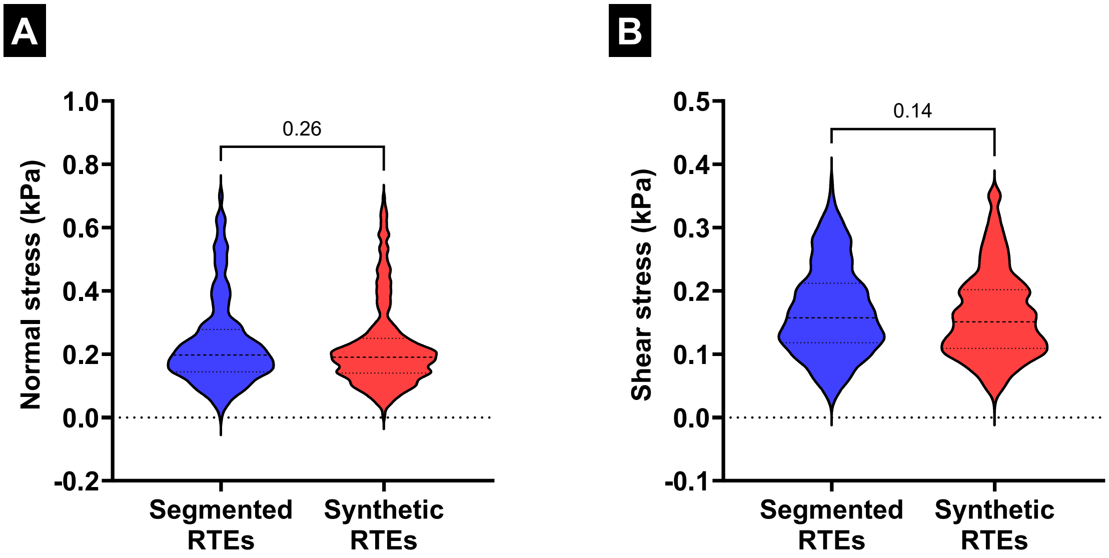

Figure S4. Comparison of (**A**) normal (**B**) shear stress between the segmented and synthetic RTEs. Statistical analysis was performed using Student's t-test.

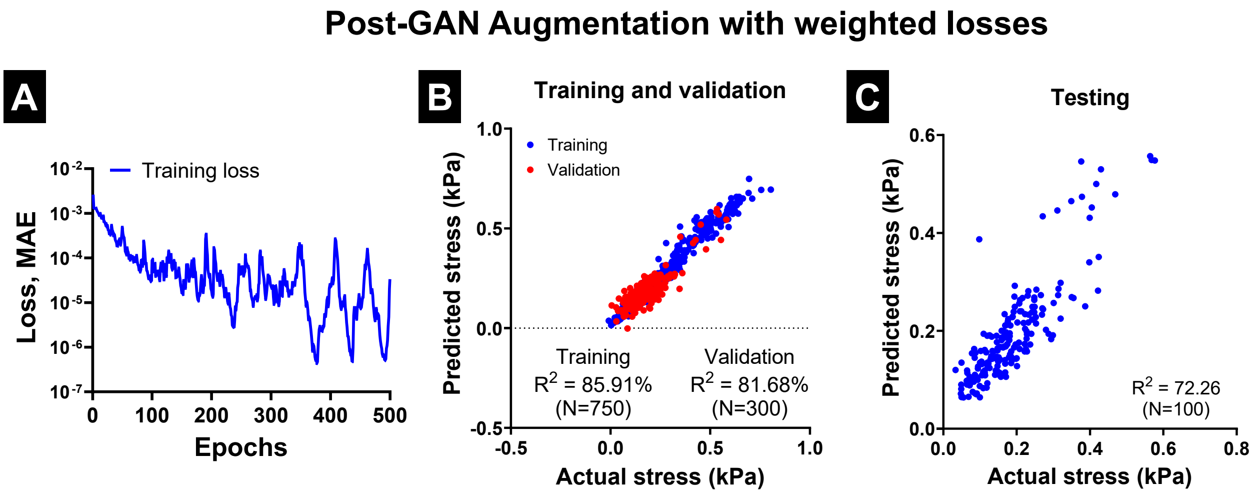

Figure S5. Training of segmented and synthetic RTEs with losses weighted to favor segmented RTEs. (**A**) Training loss (**B**) Training and validation accuracy (**C**) Testing accuracy.

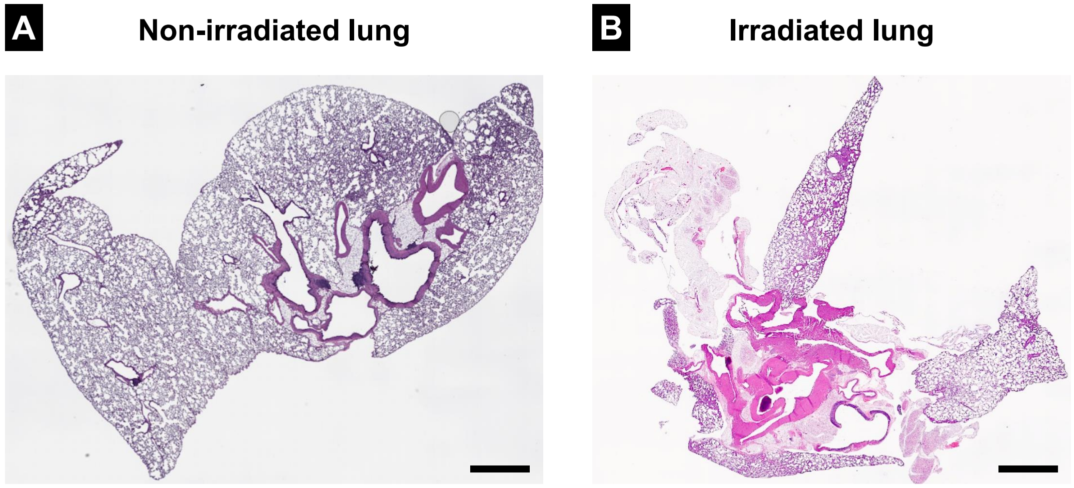

Figure S6. Representative histological images of the (**A**) non-irradiated and (**B**) irradiated lung. Scale bars in represent 1 mm

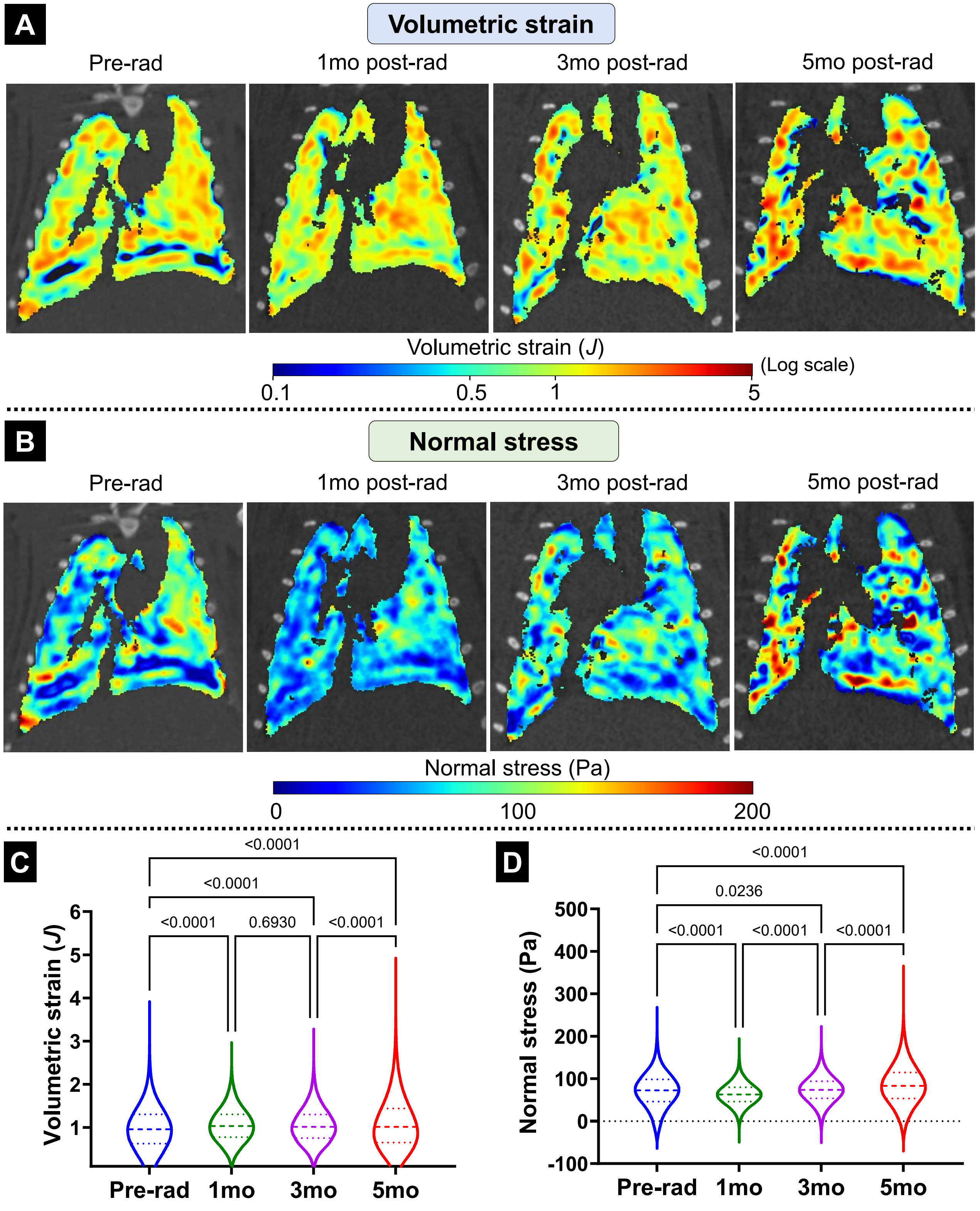

Figure S7. (**A**) Volumetric strain (J) estimated at the pre-rad, 1mo post-rad, 3mo post-rad, and 5mo post-rad timepoints. (**B**) Normal stress (σ_h) estimated at the pre-rad, 1mo post-rad, 3mo post-rad, and 5mo post-rad timepoints. Violin plots of (**C**) volumetric strain and (**D**) normal stress distribution. Statistical analysis in (**C**) and (**D**) was performed using one-way ANOVA with Tukey's correction for multiple comparisons. pre-rad: pre-radiation, mo post-rad: months post-radiation.

Table S2. Variation in normal stress in radiation induced lung injury (RILI). pre-rad: pre-radiation, mo post-rad: months post-radiation. $J > 1$: Expanding region, $J < 1$: contracting region.

Parameter	Pre-rad	1mo post-rad	3mo post-rad	5mo post-rad
J ($\forall J > 1$)	1.41 ± 0.30	1.34±0.27	1.35±0.28	1.55 ± 0.46
J ($\forall J < 1$)	0.61 ± 0.25	0.72±0.20	0.71±0.20	0.62 ± 0.25
% voxels where $J > 1$	60.6	60.7	62.0	65.1
max J	4.97	3.25	3.43	5.41
min J	0.49	0.60	0.64	0.36
mean γ_{max}	0.3±0.1	0.3±0.1	0.3±0.1	0.4±0.2
max γ_{max}	1.8	1.1	1.2	3.1
min γ_{max}	0.009	0.007	0.006	0.007
mean σ_h (Pa)	29.9±34.5	29.7±27.0	29.9±27.5	31.6±37.8
max σ_h (Pa)	520.5	477.4	483.9	518.7
min σ_h (Pa)	-60.0	-48.5	-46.9	-62.7
mean τ_{max} (Pa)	173.3±61.3	169.7±48.4	166.4±45.8	174.0±62.0
max τ_{max} (Pa)	500.7	426.6	408.7	491.7
min τ_{max} (Pa)	26.3	31.3	43.7	48.1

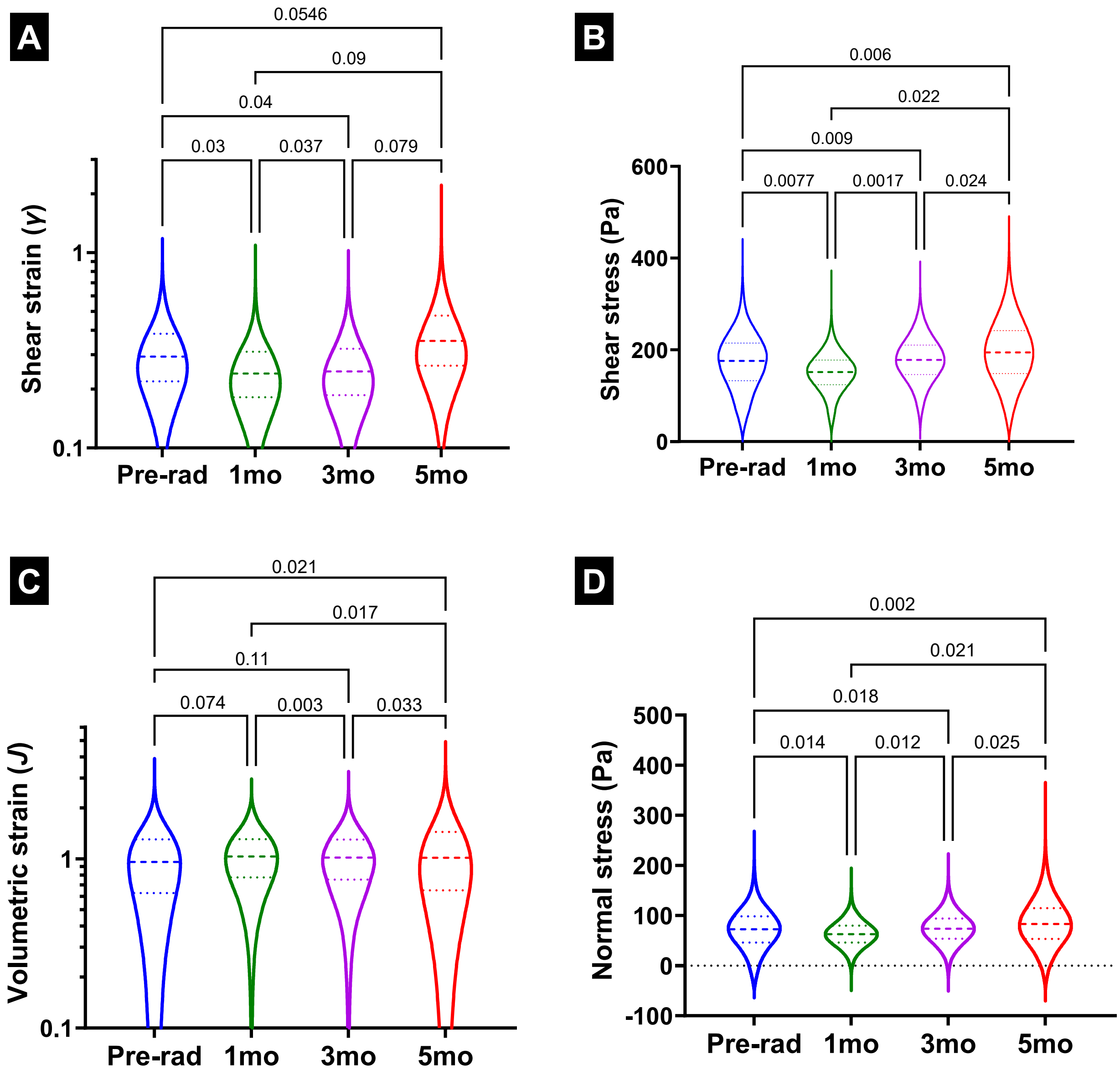

Figure S8. Violin plots of (**A**) maximum shear strain (γ_{max}) and (**D**) maximum shear stress (τ_{max}) estimated at the pre-rad, 1mo post-rad, 3mo post-rad, and 5mo post-rad timepoints. Violin plots of (**C**) Volumetric strain (J) and (**D**) Normal stress (σ_h) estimated at the pre-rad, 1mo post-rad, 3mo post-rad, and 5mo post-rad timepoints. (**A**) and (**C**) are presented in the logarithmic scale. The relative entropy between the distributions in each plot was estimated and compared using the Jensen-Shannon divergence.
